# Telemedicine in Improving Glycemic Control Among Children and Adolescents With Type 1 Diabetes Mellitus: Systematic Review and Meta-Analysis

**DOI:** 10.2196/51538

**Published:** 2024-07-09

**Authors:** Kun Zhang, Qiyuan Huang, Qiaosong Wang, Chengyang Li, Qirong Zheng, Zhuoyue Li, Dan Xu, Cuiling Xie, Mingqi Zhang, Rongjin Lin

**Affiliations:** 1 School of Nursing Fujian Medical University Fuzhou China; 2 Department of Nursing The First Affiliated Hospital of Fujian Medical University Fujian Medical University Fuzhou China; 3 Department of Infectious diseases Nanfang Hospital Southern Medical University Guangzhou China; 4 Foreign Language Department Xuzhou Medical University Xuzhou China

**Keywords:** telemedicine, digital health, web-based, type 1 diabetes mellitus, children, adolescents, glycemic control, chronic disease management, randomized controlled trials, meta-analysis, mobile phone

## Abstract

**Background:**

Type 1 diabetes mellitus (T1DM) is the most common chronic autoimmune disease among children and adolescents. Telemedicine has been widely used in the field of chronic disease management and can benefit patients with T1DM. However, existing studies lack high-level evidence related to the effectiveness of telemedicine for glycemic control in children and adolescents with T1DM.

**Objective:**

This study aims to systematically review the evidence on the effectiveness of telemedicine interventions compared with usual care on glycemic control among children and adolescents with T1DM.

**Methods:**

In this systematic review and meta-analysis, we searched PubMed, Cochrane Library, Embase, Web of Science (all databases), and CINAHL Complete from database inception to May 2023. We included randomized controlled trials (RCTs) that evaluated the effectiveness of a telemedicine intervention on glycemic control in children and adolescents with T1DM. In total, 2 independent reviewers performed the study selection and data extraction. Study quality was assessed using the Cochrane Risk of Bias 2 tool. Our primary outcome was glycated hemoglobin (HbA_1c_) levels. Secondary outcomes were quality of life, self-monitoring of blood glucose, the incidence of hypoglycemia, and cost-effectiveness. A random-effects model was used for this meta-analysis.

**Results:**

Overall, 20 RCTs (1704 participants from 12 countries) were included in the meta-analysis. Only 5% (1/20) of the studies were at high risk of bias. Compared to usual care, telemedicine was found to reduce HbA_1c_ levels by 0.22 (95% CI –0.33 to –0.10; *P*<.001; *I*^2^=35%). There was an improvement in self-monitoring of blood glucose (mean difference [MD] 0.54, 95% CI –0.72 to 1.80; *P*=.40; *I*^2^=67.8%) and the incidence of hypoglycemia (MD –0.15, 95% CI –0.57 to 0.27; *P*=.49; *I*^2^=70.7%), although this was not statistically significant. Moreover, telemedicine had no convincing effect on the Diabetes Quality of Life for Youth score (impact of diabetes: *P*=.59; worries about diabetes: *P*=.71; satisfaction with diabetes: *P*=.68), but there was a statistically significant improvement in non–youth-specific quality of life (MD –0.24, 95% CI –0.45 to –0.02; *P*=.04; *I*^2^=0%). Subgroup analyses revealed that the effect of telemedicine on HbA_1c_ levels appeared to be greater in studies involving children (MD –0.41, 95% CI –0.62 to –0.20; *P*<.001), studies that lasted <6 months (MD –0.32, 95% CI –0.48 to –0.17; *P*<.001), studies where providers used smartphone apps to communicate with patients (MD –0.37, 95% CI –0.53 to –0.21; *P*<.001), and studies with medication dose adjustment (MD –0.25, 95% CI –0.37 to –0.12; *P*<.001).

**Conclusions:**

Telemedicine can reduce HbA_1c_ levels and improve quality of life in children and adolescents with T1DM. Telemedicine should be regarded as a useful supplement to usual care to control HbA_1c_ levels and a potentially cost-effective mode. Meanwhile, researchers should develop higher-quality RCTs using large samples that focus on hard clinical outcomes, cost-effectiveness, and quality of life.

## Introduction

### Background

Type 1 diabetes mellitus (T1DM) is the most common chronic autoimmune disease among children and adolescents, characterized by hyperglycemia and caused by an absolute deficiency of insulin [1,2]. More than 1.2 million children and adolescents worldwide currently have T1DM [3]. Adolescence is a period when glycemic control commonly deteriorates [4], and people with diabetes remain at high risk of serious complications, including diabetic cardiovascular disease and diabetic nephropathy [5-7]. T1DM has a serious impact on the life health of children and adolescents. It places a heavy medical burden on the families of those affected [8,9]. Therefore, there is an imperative to explore effective treatment together with management strategies to help children and adolescents maintain normoglycemia and promote their long-term health as well as their well-being.

In recent years, telemedicine has been widely used in the field of chronic disease management. Telemedicine (a subcomponent of eHealth) has been defined as “The delivery of health care services, where distance is a critical factor, by all health care professionals using information and communications technologies for the exchange of valid information for diagnosis, treatment and prevention of disease and injuries, research and evaluation, and the continuing education of health care workers, with the aim of advancing the health of individuals and communities” [10]. For patients with chronic diseases, the advantages of telemedicine can be reflected in improving access to services, ensuring continuity of care, and mitigating the costs of care delivery [11,12]. Although telemedicine may not be able to provide physicians with comprehensive diagnostic information about a patient in the same way that a physical examination can, it can assist physicians in monitoring and recording certain specific physiological indicators (eg, blood glucose, blood oxygen concentration, blood pressure, and heart rate) to help them observe the trajectory of a patient’s health [13,14]. The current studies on telemedicine interventions for glycemic control in patients with diabetes focus on (1) telemonitoring (eg, a web-based telemedicine system was used to monitor patients with T1DM in the study by Ruiz de Adana et al [15]), (2) tele-education (eg, Molavynejad et al [16] delivered tele-education to patients with diabetes using remote video-based technology), and (3) teleconsultation and internet-based group appointments (eg, Bisno et al [17] provided both individual telehealth provider visits and internet-based group appointments for patients with T1DM through the CoYoT1 clinic). Moreover, previous meta-analyses have shown that the effectiveness of telemedicine in controlling blood glucose levels in patients with T1DM has been well validated [18-20]. It can be seen that telemedicine provides a huge advantage for diabetes glycemic control.

However, existing studies lack high-level evidence related to the effectiveness of telemedicine for glycemic control in children and adolescents with T1DM. Only a few studies have reported on the potential of telemedicine in the management of T1DM in children and adolescents. Moreover, the safety and applicability of telemedicine for children and adolescents with T1DM need to be further demonstrated. Therefore, we aimed to conduct a systematic review and meta-analysis of current randomized controlled trials (RCTs) to provide new evidence for clinical decision-making by comparing the effectiveness of telemedicine interventions with usual care in children and adolescents with T1DM.

### Study Question

How does telemedicine compare with usual care in improving glycemic control among children and adolescents with T1DM? Which form of telemedicine intervention is more effective in improving glycemic control among children and adolescents with T1DM?

### Study Objective

This meta-analysis aimed to comprehensively synthesize and evaluate evidence on the effectiveness of telemedicine on glycemic control among children and adolescents with T1DM.

## Methods

### Search Strategy

In total, 5 electronic databases covering the realms of biomedicine science, clinical medicine science, and general references were screened: PubMed, Cochrane Library, Embase, Web of Science (all databases), and CINAHL Complete. The dates searched were from establishment of each database to May 1, 2023. The search was conducted using the following keywords: (“Diabetes Mellitus, Type 1”) AND (“Telemedicine” OR “Telemetry” OR “Telenursing” OR “Internet-Based Intervention”) AND (“Child” OR “Adolescent”). Medical Subject Heading terms and their related terms were used. Multimedia Appendix 1 [21-40] shows the detailed search terms and search process. There were no restrictions in terms of participant age, year of publication, or region of study at this stage. The review protocol was reported according to the PRISMA (Preferred Reporting Items for Systematic Reviews and Meta-Analyses) 2020 checklist (Multimedia Appendix 2).

### Inclusion and Exclusion Criteria

The inclusion criteria were defined by population, intervention, comparison, outcome, and study design as follows:

1. Population: the target participants were children (aged ≤10 years) and adolescents (10 years<age≤19 years) [41] with T1DM.
2. Intervention: complete or partial telemedicine intervention. A complete telemedicine intervention was one in which there was no face-to-face contact between the participants and the health care providers throughout the trial period from baseline to the end of the intervention and the only telemedicine interventions were via telephone, web-based videoconferencing, a website, or a smartphone app (all treatments [including initial treatment] were delivered via telemedicine). *Partial telemedicine intervention* referred to treatments that combine telemedicine with nontelemedicine interventions (such as a follow-up visit in an outpatient clinic or a visit at home). These 2 broad categories of telemedicine interventions were further subdivided by the number of intervention forms. “Single” refers to the inclusion of only 1 form of telemedicine intervention, whereas “mixed” refers to the inclusion of ≥2 forms of telemedicine intervention. Complete telemedicine interventions were categorized as single and mixed complete telemedicine interventions; partial telemedicine interventions were categorized as single and mixed partial telemedicine interventions.
3. Comparison: containing a comparison group with usual care, including a nontelemedicine intervention and health guidance only before discharge treated as a blank control.
4. Outcome: we included all studies that reported serum glycated hemoglobin (HbA_1c_) levels as either their primary or secondary outcomes.
5. Study design: only RCTs (parallel or crossover) were included.

The exclusion criteria were (1) studies using nonexperimental and quasi-experimental designs; (2) abstracts, brief reports, conference proceedings, conference papers, posters, and letters to editors; (3) studies on patients with gestational diabetes; and (4) studies published in languages other than English because of our lack of high-quality translational resources.

### Study Screening

Throughout the screening processes, all studies included in the analysis were independently reviewed by 2 researchers (KZ and CL). First, we screened the titles and abstracts of all bibliographic records against the inclusion and exclusion criteria, and a label was created on a serial numbered sheet to add the reason for exclusion as a note. Second, we thoroughly read the full text of the study without exclusion labels to ensure that all inclusion and exclusion criteria were met. Disagreements between the researchers were resolved by meeting with a third reviewer (QH). Studies judged to be eligible at this stage were then included in the quality assessment where applicable.

### Quality Assessment

We assessed the risk of bias using the Cochrane Risk of Bias 2 tool [42] to evaluate the randomization process, deviations from the intended interventions, missing outcome data, measurement of the outcome, and selection of the reported results. In total, 2 researchers (KZ and CL) assessed the trials independently and resolved any disagreements by meeting with a third reviewer (QH). The quality of evidence of each study was assessed by 2 reviewers (QH and QW) using the Grading of Recommendations, Assessment, Development, and Evaluations approach [43].

### Outcome

The primary outcome was HbA_1c_ levels. Secondary outcomes were quality of life as measured using a validated instrument, daily frequency of self-monitoring of blood glucose (SMBG), the incidence of hypoglycemia, and cost-effectiveness.

### Data Extraction

For each included study, 2 reviewers (KZ and CL) independently extracted the data for analysis. When data were missing or unclear, we contacted the authors. If the authors did not respond, the study was reassessed and excluded.

We extracted the following information from the selected studies: (1) study characteristics (study name, author, year of publication, country, study design, attrition rate, and sample size), (2) characteristics of the participants (age, gender, diabetes duration, baseline HbA_1c_ levels, total cholesterol levels, triglyceride levels, blood pressure, and BMI), (3) intervention details (duration, types of health care providers, frequency of feedback, characteristics of intervention content, communication forms between providers and patients, technology use modes, and telemedicine intervention forms; communication forms included modem, SMS text messaging, email, web conference, website—websites where patients upload blood glucose levels or other clinical data and share them with their health care providers—computer software, smart wearable devices—smart wearable devices are consumer-grade connected electronic devices that can be worn on the body as an accessory or embedded into clothing [44] —telephone, and smartphone or its apps), and (4) general information about outcomes (the mean and SD at baseline and at the end of the intervention, number of participants analyzed at the end of the intervention, and tools used for measurement; when several analyses were performed on the same outcome at the same time point, we extracted the data from the intention-to-treat analysis).

### Data Analysis

Stata (version 17; StataCorp) and Review Manager (version 5.4; The Cochrane Collaboration) were used for all statistical analysis. For quantitative synthesis, we collected the difference between baseline and end-point values for both the intervention and control groups. In the absence of information, data were estimated from the mean and SD of baseline and end-point values using a correlation of 0.5 [45]. To ensure accuracy, different correlations, such as 0.4 and 0.6, were used for estimation data and sensitivity analysis. The final results showed that the estimated results obtained using the different correlations remained stable after sensitivity analysis [45]. Data conversion tools were used to convert the median, maximum, and minimum values reported in the included studies into mean [46] and SD [47]. We reported the results of secondary outcomes when data from at least 2 studies could be merged. The magnitude of the overall effect size was calculated based on the pooled mean difference (MD) with 95% CI when the same measures were used in the studies. If outcomes were measured using different outcome measurement scales, the pooled standardized MD (SMD) with 95% CI was adopted. A *P* value of <.05 was considered statistically significant.

A random-effects or fixed-effects meta-analysis for continuous data was performed based on the results of the heterogeneity test. Study heterogeneity was determined using the Cochran *Q* test and Higgins *I*^2^ test. *I*^2^ values of 25%, 50%, and 75% indicated low, moderate, and high heterogeneity, respectively [45]. If *P*>.10 and *I*^2^<50% were identified, fixed-effects models were used; otherwise, random-effects models were applied. To ensure the robustness of our results, a sensitivity analysis was performed by using leave-one-out analysis to assess the contribution of each study to the merged effect size.

Publication bias was assessed creating funnel plots, the Begg test, and performing the Egger regression test (considered significant at *P<*.05) by 2 reviewers (QW and CL), and agreement was reached through consensus [48,49]. For the primary outcome, we performed a series of subgroup analyses to quantify specific differences in the size of effects of particular telemedicine interventions based on study and intervention characteristics [50]. Moreover, we performed a univariable meta-regression analysis to investigate whether there was heterogeneity due to differences in study or intervention characteristics.

### Protocol Deviation

First, the definition of the intervention group in the registration program as an Internet-based telemedicine intervention group is too broad and simplistic. After further research, we decided to categorize the interventions into complete and partial telemedicine interventions. These 2 broad categories of telemedicine interventions were further subdivided by the number of intervention forms. “Single” refers to the inclusion of only 1 form of telemedicine intervention, whereas “mixed” refers to the inclusion of ≥2 forms of telemedicine intervention. Complete telemedicine interventions were categorized as single and mixed complete telemedicine interventions; partial telemedicine interventions were categorized as single and mixed partial telemedicine interventions. These changes and clarifications help explain the impact of the “face-to-face contact between patient and healthcare provider” factor on telemedicine effectiveness during telemedicine interventions, which has important implications for the development of future telemedicine interventions.

Second, the definition of the control group (“usual care”) was also an oversimplification, so we illustrated 2 cases of “usual care” in this study, including a nontelemedicine intervention as well as health guidance only before discharge treated as a blank control.

Third, regarding secondary outcomes, initially, we identified secondary outcomes based on studies related to diabetes telemedicine in adults and other types of diabetes. However, during the literature reading, it was found that no studies analyzed blood pressure, weight, and patient satisfaction as study outcomes in telemedicine interventions on children and adolescents with T1DM. Some studies used only weight and blood pressure as baseline indicators and lacked postintervention data. Other studies only asked participants how satisfied they were with the telemedicine intervention through interviews at the end of the intervention, which prevented us from quantitatively assessing satisfaction. Therefore, secondary outcomes such as blood pressure, weight, and patient satisfaction were removed.

Finally, regarding the data synthesis strategy, we modified the section for missing data estimation. Because data for the primary and secondary outcomes were partially missing, we first used the commonly used correlation coefficient of 0.5 for data estimation according to the Cochrane Handbook for Systematic Reviews of Interventions [45]. However, as there is currently no clear specification for the use of correlation coefficients for data estimation (only a broad range of choices), to ensure that the effect sizes synthesized using the “estimated data” were sufficiently stable, we also used 0.4 and 0.6 as correlation coefficients for data estimation. (Our main purpose was to see whether the estimated effects using the new correlation coefficients would pass the sensitivity analyses after changing the correlation coefficient). The sensitivity analyses showed that the results synthesized after estimating the missing data using all 3 correlation coefficients were stable and reliable, but the data estimated using the more common correlation coefficient of 0.5 was used as the results of this study.

## Results

### Search Results

**Figure 1:**
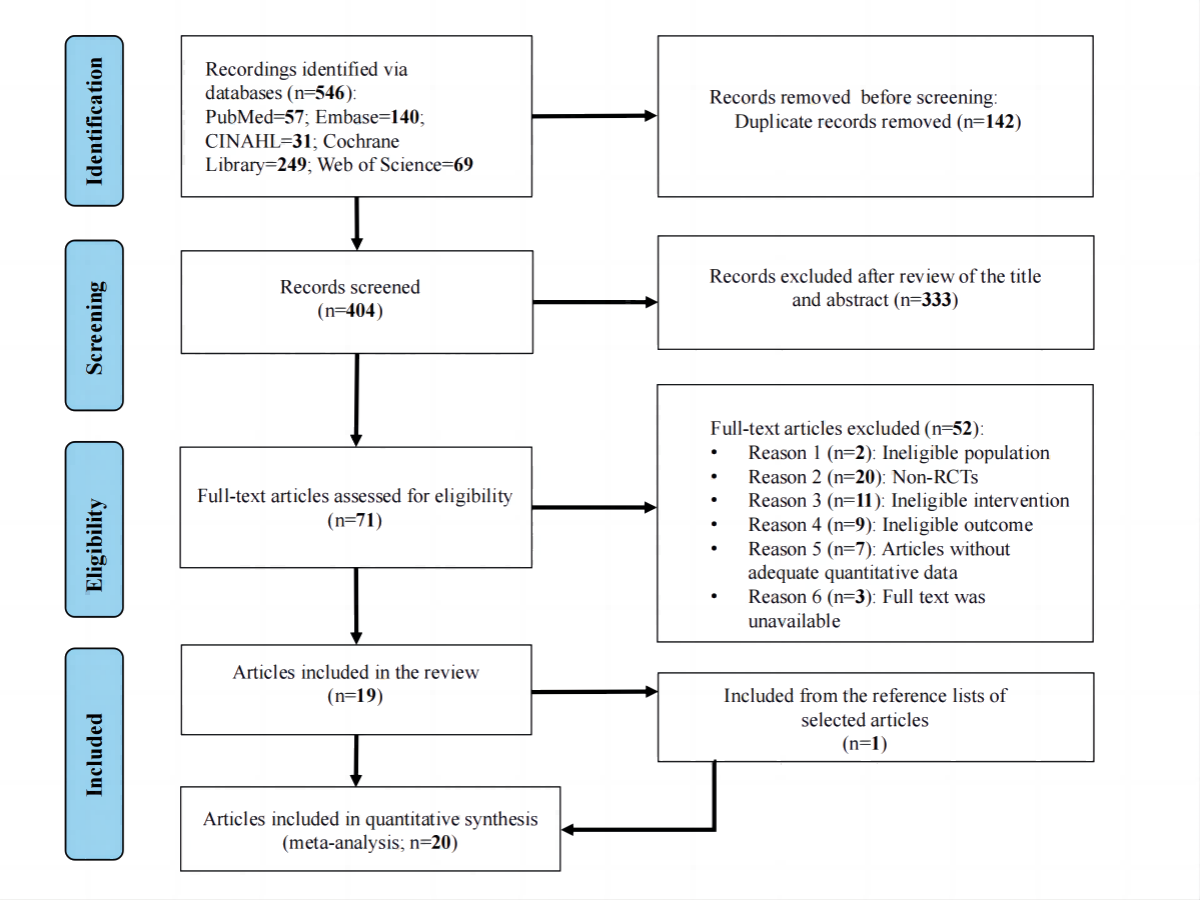
PRISMA (Preferred Reporting Items for Systematic Reviews and Meta-Analyses) flowchart depicting the main stages of the systematic review process. RCT: randomized controlled trial.

The phases of electronic search, identification, and screening for eligible studies are depicted in the PRISMA flowchart (Figure 1). A total of 546 studies were identified using the search strategy described previously. After removing duplicates and screening titles and abstracts, a total of 20 studies were retained for full-text evaluation. Finally, a total of 20 studies with 1704 participants were included in this systematic review and meta-analysis.

### Study Characteristics

The characteristics of the studies are summarized in Table S1 in Multimedia Appendix 1. A total of 90% (18/20) of the studies were parallel-group RCTs [21-38], and 10% (2/20) were crossover studies [39,40]. Of the 20 included studies, 12 (60%) were published after 2015. In total, 40% (8/20) of the studies were published in North America [21-24,27,29,30,34], 35% (7/20) were published in Europe [25,26,33,35-37,39], 20% (4/20) were published in Asia [28,32,38,40], and 5% (1/20) were published in Oceania [31]. The sample sizes of the studies ranged from 20 to 240, with the intervention periods ranging from 3 to 60 months. All participants included in the studies were aged <20 years and had T1DM. The median mean age at baseline was 13.5 years, and the median mean diabetes duration at baseline was 6.2 years. A total of 90% (18/20) of the studies were performed in adolescents (mean age 13.6; range 10.8-17.3 years), and 10% (2/20) of the studies were performed in children (mean age 5.8; range 5.6-6.1 years). The proportion of female participants at baseline ranged from 42% to 62%. The floored threshold value of baseline HbA_1c_ levels in 35% (7/20) of the studies was ≥7.5%.

### Intervention Characteristics

The telemedicine systems used in most studies were relatively simple to operate, having clear processes and including transmission of blood glucose data with feedback (15/20, 75%) [21-24,26,28,29,31,33-37,39,40] or blood glucose data only (5/20, 25%) [25,27,30,33,38]. A specialist diabetes care team, including a diabetologist, nurse, dietician, and psychologist, was reported in 45% (9/20) of the studies [22,24,29,31,33-35,37,40]. Feedback was provided monthly or less frequently in 50% (10/20) of the studies [23,26,31,33-37,39,40] and every 2 weeks or more frequently in 25% (5/20) of the studies [21,22,24,28,29], and the frequency of feedback was not specified in 25% (5/20) of the studies (Table 1) [25,27,30,32,38].

**Table 1:** Characteristics of the telemedicine interventions.

Study, year, and country or region	Health care provider	Communication form			Frequency of feedback		Intervention content						Technology use mode		Technology form		Telemedicine intervention form
		Provider to patient	Patient to provider			Internet-based follow-up		Medication adjustment	Diet guidance	Physical exercise	Basic health education						
Chase et al [21], 2003, United States	Nurse and physician	Telephone	Modem	Every 2 weeks		Yes		No	No	No	Yes	—^a^		Hardware		Complete telemedicine intervention (single)	
Gandrud et al [22], 2018, United States	Diabetes educator, nurse, and physician	SMS text messaging and email	Smartphone app	Weekly		Yes		Yes	No	Yes	Yes	—		Software		Partial telemedicine intervention (mixed)	
Goyal et al [23], 2017, Canada	Human factors specialist, nurse, and physician	Smartphone app and telephone	Smart wearable device	Every 3 months		Yes		No	No	No	No	Independently		Software		Partial telemedicine intervention (mixed)	
Han et al [24], 2015, United States	Diabetes educator, nurse, and physician	SMS text messaging	Smartphone app and SMS text messaging	Every 2 days		Yes		Yes	No	No	Yes	Independently		Software		Complete telemedicine intervention (single)	
Ibrahim et al [25], 2021, Europe	Diabetologist	SMS text messaging	—	—		No		No	No	No	No	Independently		Software		Partial telemedicine intervention (single)	
Klee et al [39], 2018, Switzerland	Nurse and diabetologist	Smartphone app and website	Telephone and email	Monthly		Yes		Yes	Yes	No	No	—		Software		Partial telemedicine intervention (mixed)	
Kowalska et al [26], 2017, Poland	Pediatrician and diabetologist	Computer software	Smart wearable device	Every 13 weeks		Yes		Yes	Yes	No	No	Parental assistance		Software		Partial telemedicine intervention (single)	
Kumar et al [27], 2004, United States	Trained research assistant	Website	Modem and smart wearable device	—		No		Yes	Yes	No	No	Parental assistance		Software		Complete telemedicine intervention (single)	
Landau et al [28], 2012, Israel	Dietitian and pediatric endocrinologist	Telephone	Website and smart wearable device	Every week		Yes		Yes	No	No	No	Independently		Software		Partial telemedicine intervention (mixed)	
Marrero et al [29], 1995, United States	Pediatric diabetologist, nurse, social workers, and dietitians	Computer software and telephone	Modem	Every 2 weeks		Yes		Yes	Yes	No	No	—		Software		Partial telemedicine intervention (mixed)	
Mulvaney et al [30], 2010, United States	Diabetes professionals	Website	Website	—		No		No	No	No	Yes	—		Software		Complete telemedicine intervention (single)	
Nunn et al [31], 2006, Australia	Pediatric endocrinologists, nurse, dietitian, and social worker	Telephone	Telephone	Every 2 months		Yes		Yes	Yes	Yes	Yes	Parental assistance		Hardware		Complete telemedicine intervention (single)	
Raviteja et al [32], 2019, India	Consultant and physician	Smart wearable device	Smart wearable device and computer software	—		Yes		Yes	Yes	Yes	No	Independently		Hardware		Complete telemedicine intervention (mixed)	
Schiaffini et al [33], 2016, Italy	Diabetologist, nurse, dietician, and psychologist	Web conference	Website and smart wearable device	Every month		Yes		No	Yes	Yes	Yes	Parental assistance		Software		Complete telemedicine intervention (mixed)	
Shalitin et al [40], 2014, Israel	Diabetes care team	Website, email, and telephone	Smart wearable device and website	Every month		Yes		Yes	No	No	No	Parental assistance		Software		Complete telemedicine intervention (mixed)	
Stanger et al [34], 2018, United States	Pediatric endocrinologist and diabetes care team	Web conference	Smart wearable device	Every month (last period)		Yes		Yes	No	No	No	Parental assistance		Software		Complete telemedicine intervention (single)	
Von Sengbusch et al [35], 2020, Germany	Regular home diabetes team	Web conference	Smart wearable device and computer software	Every month		Yes		Yes	No	Yes	Yes	Parental assistance		Software		Partial telemedicine intervention (single)	
Ware et al [36], 2022, United Kingdom	Nurse and physician	Smartphone app, telephone, and email	Smartphone app and smart wearable device	Every month		Yes		Yes	No	No	No	Parental assistance		Software		Complete telemedicine intervention (mixed)	
Ware et al [37], 2022, United Kingdom	Research team and clinical team	Smartphone app, telephone, and email	Smartphone app and smart wearable device	Every month		Yes		Yes	Yes	No	Yes	Parental assistance		Software		Complete telemedicine intervention (mixed)	
Xu et al [38], 2021, China	Nurse and third-party health manager	Smartphone app	Smartphone app and smart wearable device	—		Yes		No	Yes	Yes	Yes	Independently		Software		Complete telemedicine intervention (mixed)	

^a^Not reported.

The communication technologies used in the telemedicine interventions included in the studies took a variety of forms. Patients initiated communication with health care providers through different forms of telemedicine: smart wearable devices (6/20, 30%) [23,26,32,34,35,40], smartphone apps (5/20, 25%) [22,24,36-38], modem (3/20, 15%) [21,27,29], websites (3/20, 15%) [28,30,33], telephone (2/20, 10%) [31,39], and unclear (1/20, 5%) [25]. Health care providers initiated communication with patients through different forms of telemedicine: smartphone apps (5/20, 25%) [23,36-39], websites (3/20, 15%) [27,30,40], web conferences (3/20, 15%) [33-35], telephone (3/20, 15%) [21,28,31], SMS text messaging (3/20, 15%) [22,24,25], computer software (2/20, 10%) [26,29], or smart wearable devices (1/20, 5%) [32]. A total of 85% (17/20) of the studies mainly used various types of software [22-30,33-40], and 15% (3/20) of the studies used hardware [21,31,32].

In total, 45% (9/20) of the studies involved patients using telemedicine with parental assistance [26,27,31,33-37,40], and 30% (6/20) of the studies involved patients using telemedicine independently [23-25,28,32,38]. The form of intervention was complete telemedicine intervention in 60% (12/20) of the studies [21,24,27,30-34,36-38,40] and partial telemedicine intervention in 40% (8/20) of the studies [22,23,25,26,28,29,35,39]. The content of the telemedicine interventions in the studies included a variety of features: internet-based communication and follow-up (17/20, 85%) [21-24,26,28,29,31-40], medication dose adjustment (14/20, 70%) [22,24,26-29,31,32,34-37,39,40], basic health education (9/20, 45%) [21,22,24,30,31,33,35,37,38], diet guidance (9/20, 45%) [26,27,29,31-33,37-39], and physical exercise (6/20, 30%) [22,31-33,35,38]. A total of 55% (11/20) of the studies reported characteristics of the content of the intervention including at least 3 features [22,24,26,29,31-33,35,37-39]. No features of the content of the telemedicine interventions were reported in 5% (1/20) of the studies [25].

### Risk of Bias

**Figure 2:**
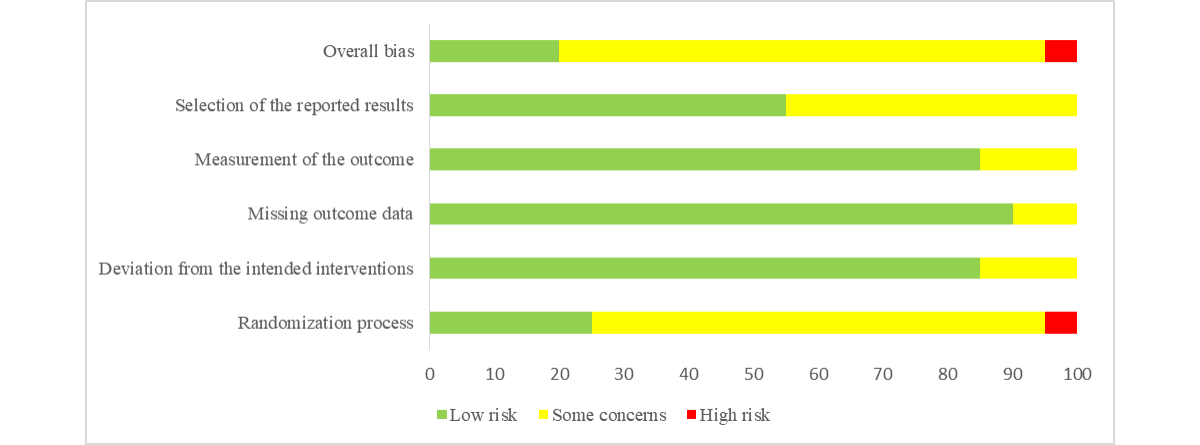
Risk-of-bias graph of the included studies (part 1).

**Figure 3:**
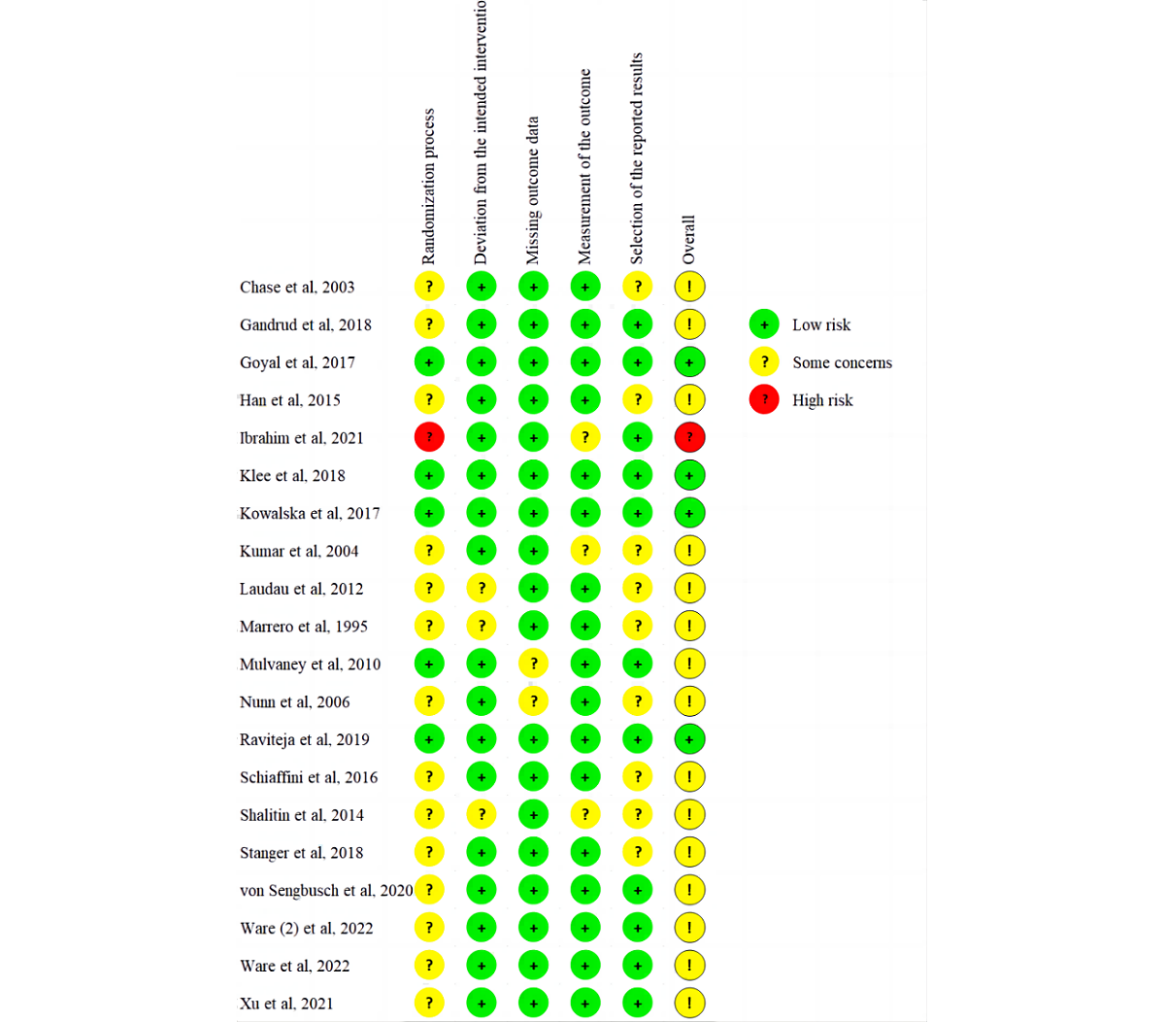
Risk-of-bias graph of the included studies (part 2).

On the basis of the Cochrane Risk of Bias 2 tool, all studies except for 5% (1/20) with a high risk of bias [25] and 20% (4/20) with a low risk of bias [23,26,32,39] were found to have “some concerns” (Figures 2 and 3 [21-40]). The greatest bias was found in the randomization process. Randomization was reported to be implemented in all studies, among which only 25% (5/20) of the studies explicitly described the randomization strategies and properly applied allocation concealment [23,26,30,32,39]. The other study [25] was rated as high risk because of baseline differences between intervention groups. No preregistration was reported in 45% (9/20) of the studies [21,24,27-29,31,33,34,40], and the risk of bias regarding the choice of reporting outcomes was rated as “some concerns.” One of the domains with the highest proportion of low risk of bias was “bias from missing outcome data.”

### Meta-Analysis and Descriptive Analysis Results

**Figure 4:**
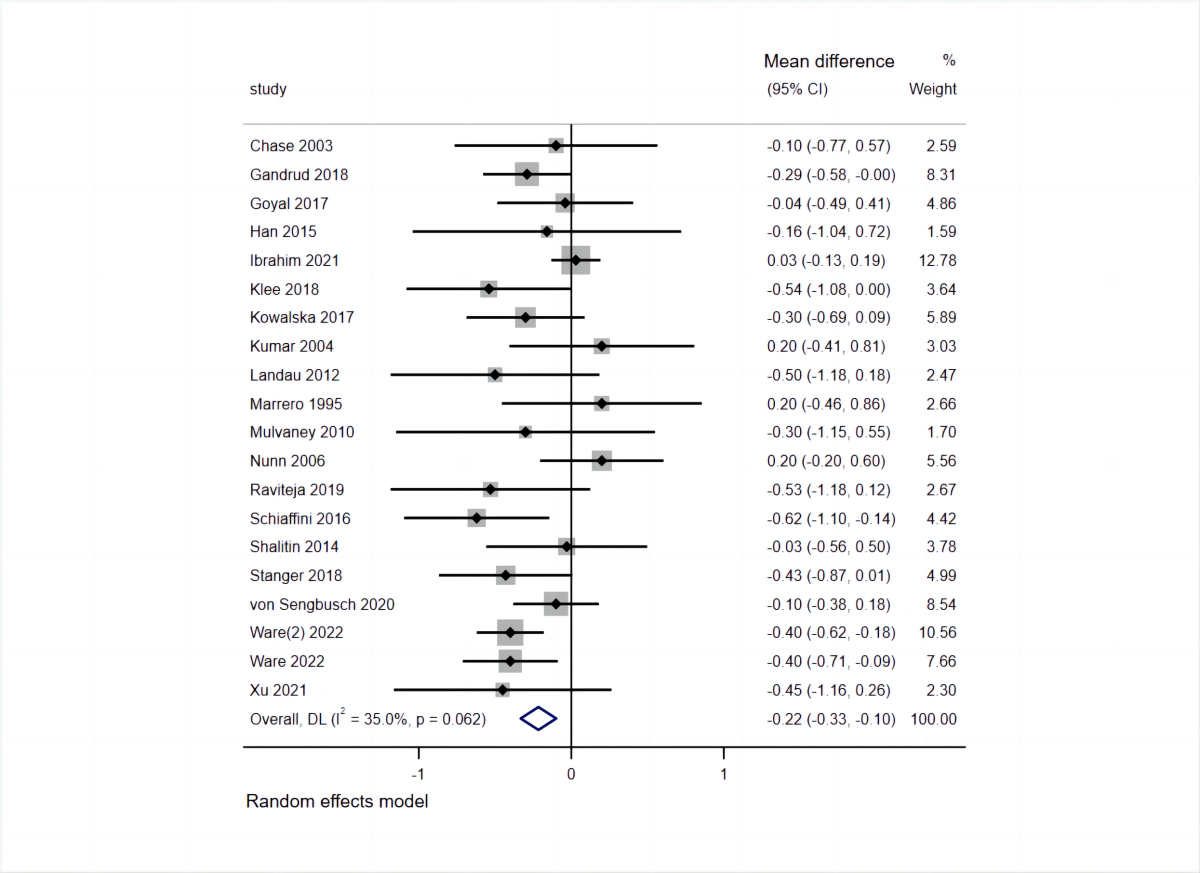
Forest plot of the comparison of telemedicine interventions versus usual care. Outcome: glycated hemoglobin. DL: DerSimonian and Laird approach.

A summary of the main results for the comparisons using the Grading of Recommendations, Assessment, Development, and Evaluations ratings is presented in Table 2. Detailed meta-analytic forest plots on all outcomes and subgroups are shown in Figure 4 and Figure S1 in Multimedia Appendix 1.

**Table 2:** Summary of findings—telemedicine compared to usual care for glycemic control in children and adolescents with type 1 diabetes mellitus.

Certainty assessment									Patients, n			Effect, absolute (95% CI; *P* value)		Certainty
Studies, n (%)		Study design	Risk of bias	Inconsistency	Indirectness	Imprecision	Other considerations	Telemedicine		Usual care				
**HbA_**1c**_^a^**														
	20 (100)	Randomized trials	Serious^b^	Not serious^c^	Not serious	Serious^d^	None	822		822	MD^e^ –0.22 (–0.33 to –0.10; <.001)		Low	
**DQOLY^f^ (impact of diabetes)**														
	2 (10)	Randomized trials	Serious^g^	Not serious	Not serious	Very serious^h^	None	30		23	MD 1.27 (–3.31 to 5.86; .59)		Very low	
**DQOLY (worries about diabetes)**														
	2 (10)	Randomized trials	Serious^g^	Not serious	Not serious	Very serious^h^	None	30		23	MD 0.58 (–2.59 to 3.66; .71)		Very low	
**DQOLY (satisfaction with diabetes)**														
	2 (10)	Randomized trials	Serious^g^	Serious^i^	Not serious	Very serious^h^	None	30		23	MD 3.27 (–12.53 to 19.08; .68)		Very low	
**N-QOL^j^**														
	3 (15)	Randomized trials	Serious^k^	Not serious	Not serious	Serious^l^	None	165		160	SMD^m^ –0.24 (–0.45 to –0.02; .04)		Low	
**SMBG^n^**														
	3 (15)	Randomized trials	Serious^o^	Serious^i^	Not serious	Very serious^h^	None	96		91	MD 0.54 (–0.72 to 1.8; .40)		Very low	
**Incidence of hypoglycemia**														
	4 (20)	Randomized trials	Serious^p^	Serious^i^	Not serious	Serious^l^	None	153		156	MD **–**0.22 (–0.66 to 0.23; .49)		Very low	

^a^HbA_1c_: glycated hemoglobin.

^b^Downgraded for unclear or inadequate randomization process (15/20, 75% of the included studies). In a large number of studies, allocation was not adequately concealed due to the nature of the intervention.

^c^Although the Cochran *Q* test and Higgins *I*^2^ test suggested a low heterogeneity, we chose not to downgrade for inconsistency as this was fully explained by the inclusion of 1 study.

^d^A total of 65% (13/20) of the studies had sample sizes of <50 in both arms.

^e^MD: mean difference.

^f^DQOLY: Diabetes Quality of Life for Youth.

^g^One of the studies had some concerns (a moderate risk of bias).

^h^Sample sizes for each arm of the included studies were <50.

^i^Significant heterogeneity.

^j^N-QOL: non–youth-specific quality of life.

^k^All 3 studies had some concerns.

^l^There was at least 1 study with a sample size of <50 in both arms.

^m^SMD: standardized mean difference.

^n^SMBG: self-monitoring of blood glucose.

^o^A total of 10% (2/20) of the studies had some concerns.

^p^One study had some concerns.

### Effect of Telemedicine Interventions on HbA1c

The 20 studies, which reported HbA_1c_ levels at 3 to 50 months and examined 1704 participants, were included in the meta-analysis. Overall, telemedicine was found to reduce HbA_1c_ levels by 0.22 (95% CI –0.33 to –0.10; *P*<.001) at the end of the intervention. Furthermore, the heterogeneity of the effect size was confirmed as *I*^2^ was 35% (*Q*_19_=29.23; *P*=.06), suggesting heterogeneity of a low degree. Given the wide variety of technologies available for telemedicine, the heterogeneity of results is not surprising. No significant improvements were noted at the end of the 3- (MD –0.30, 95% CI –0.62 to 0.02; *P*=.07; n=4) or 12-month (MD –0.04, 95% CI –0.33 to 0.40; *P*=.85; n=2) follow-up; however, significant improvement was found at the end of the 6-month follow-up (MD –0.21, 95% CI –0.37 to –0.05; *P*=.01; n=8).

### Effect of Telemedicine Interventions on Secondary Outcomes

We pooled the Diabetes Quality of Life for Youth (DQOLY) scores [51,52] from 10% (2/20) of the studies (n=53) [24,39], non–youth-specific quality of life (N-QOL; using diabetes quality of life [53] and health-related quality of life [54]) from 15% (3/20) of the studies (n=334) [35,38,40], daily frequency of SMBG from 15% (3/20) of the studies (n=187) [23,34,40], and incidence of hypoglycemia from 20% (4/20) of the studies (n=309) [23,26,32,38].

There was no significant effect size in secondary outcomes except for the N-QOL, with MD for DQOLY (DQOLY impact of diabetes subscale: MD 1.27, 95% CI –3.31 to 5.86, n=53, and *I*^2^=32.2%; DQOLY worries about diabetes subscale: MD 0.58, 95% CI –2.49 to 3.66, n=53, and *I*^2^=23.8%; DQOLY satisfaction with diabetes subscale: MD 3.27, 95% CI –12.53 to 19.08, n=53, and *I*^2^=75.6%), an SMD of −0.24 for the N-QOL (95% CI –0.45 to –0.02; n=334; *I*^2^=0%), an MD of 0.54 for daily frequency of SMBG (95% CI –0.72 to 1.80; n=187; *I*^2^=67.8%), and an SMD of −0.22 for incidence of hypoglycemia (95% CI –0.66 to 0.23; n=309; *I*^2^=73.7%).

Only 5% (1/20) of the studies [21] reported economic data. The difference in cost-effectiveness of care between the 2 groups was significant. The average cost per patient in the intervention group for the 6 months was US $163. The control group spent an average of US $246 to visit the clinic. If additional costs (average US $59), such as mileage, parking, meals, hotel stays, and babysitting, were included, the average cost of a clinic visit increased to US $305. This result shows that the telemedicine intervention was cost-effective, at least in the United States.

### Subgroup Analysis of HbA1c

Our subgroup analysis based on study and intervention characteristics revealed that the subgroup differences that yielded statistical significance were publication date, communication forms (from patient to provider), and internet-based follow-up (Table 3).

Regardless of age, intervention duration, and health care provider, HbA_1c_ levels significantly decreased in all studies after the telemedicine intervention.

**Table 3:** Summary of subgroup analysis based on glycated hemoglobin.

Characteristic and subgroup				Number of trials (number of participants)		Effect size, MD^a^ (95% CI)		*I*^2^ (%)		*P* value (*Q* test)		Heterogeneity between groups		
**Age**														0.058
	Children		2 (210)		–0.41 (–0.62 to –0.20)		0		.71					
	Adolescents		18 (1434)		–0.18 (–0.30 to –0.06)		28.1		.13					
**Publication date**														0.010
	2010 and before		5 (384)		0.11 (–0.15 to 0.37)		0		.80					
	After 2010		15 (1260)		–0.27 (–0.40 to –0.15)		36.8		.08					
**Intervention duration**														0.199
	<6 months		8 (495)		–0.32 (–0.48 to –0.17)		0		.58					
	At least 6 months		12 (1149)		–0.18 (–0.33 to –0.03)		43.2		.06					
**Health care provider**														0.884
	Professional diabetes care team		9 (850)		–0.21 (–0.38 to –0.04)		33.5		.15					
	No professional diabetes care team		11 (794)		–0.23 (–0.40 to –0.06)		40.7		.08					
**Feedback frequency**														0.426
	More than once a month		5 (364)		–0.23 (–0.449 to –0.002)		0		.63					
	Less than or equal to once a month		10 (983)		–0.27 (–0.41 to –0.12)		35.6		.12					
	Unclear		5 (297)		–0.08 (–0.32 to 0.16)		20.3		.29					
**Communication form**														
	**Provider to patient**												0.259	
		Telephone	3 (244)		–0.06 (–0.46 to 0.35)		34.9		.22					
		SMS text messaging	3 (229)		–0.10 (–0.35 to 0.15)		45.4		.16					
		Smartphone app	5 (440)		–0.37 (–0.53 to –0.21)		0		.62					
		Computer software	2 (211)		–0.12 (–0.59 to 0.35)		39.6		.20					
		Website	3 (127)		0.00 (–0.36 to 0.36)		0		.63					
		Web conference	3 (330)		–0.34 (–0.65 to –0.02)		49.9		.14					
		Smart wearable device	1 (63)		−0.53 (−1.18 to 0.12)		—^b^		—					
	**Patient to provider**												0.002	
		Modem	3 (209)		0.11 (–0.26 to 0.48)		0		.76					
		Smartphone app	5 (453)		–0.37 (–0.51 to –0.22)		0		.96					
		Smart wearable device	6 (595)		–0.20 (–0.37 to –0.03)		0		.60					
		Telephone	2 (156)		–0.15 (–0.87 to 0.58)		78.3		.03					
		Website	3 (139)		–0.53 (–0.89 to –0.18)		0		.81					
		Not reported	1 (92)		0.03 (–0.13 to 0.19)		—		—					
**Technology form**														0.505
	Hardware		3 (249)		–0.08 (–0.51 to 0.35)		43.2		.17					
	Software		17 (1395)		–0.23 (–0.36 to –0.11)		34.8		.08					
**Technology use mode**														0.534
	Independent use		6 (374)		–0.11 (–0.31 to 0.09)		0		.53					
	Parental assistance		9 (899)		–0.24 (–0.41 to –0.07)		44.1		.07					
	Unclear		5 (371)		–0.26 (–0.47 to –0.04)		0		.53					
**Telemedicine intervention form**														0.206
	Complete telemedicine intervention		12 (802)		–0.28 (–0.43 to –0.13)		20.5		.24					
	Partial telemedicine intervention		8 (842)		–0.14 (–0.29 to 0.01)		29.4		.19					
**Internet-based follow-up**														0.002
	With feature		17 (1460)		–0.27 (–0.38 to –0.17)		8.9		.35					
	Without feature		3 (184)		0.03 (–0.12 to 0.18)		0		.64					
**Medication adjustment**														0.577
	With feature		14 (1267)		–0.25 (–0.37 to –0.12)		20		.24					
	Without feature		6 (377)		–0.17 (–0.41 to 0.07)		37.4		.16					
**Physical exercise**														0.823
	With feature		6 (691)		–0.24 (–0.47 to 0.01)		44.6		.11					
	Without feature		14 (953)		–0.21 (–0.35 to –0.07)		35.2		.09					

^a^MD: mean difference.

^b^Data synthesis is not possible with only one study.

### Population of the Study

No statistically significant subgroup differences were identified in the subgroup analysis by age. A statistically significant decrease in HbA_1c_ levels was observed in subgroups of children (MD –0.41, 95% CI –0.62 to –0.20; *P*<.001) and adolescents (MD –0.18, 95% CI –0.30 to –0.06; *P*=.003). The children subgroup reported a higher MD than the adolescent subgroup.

### Publication Date of the Studies

Subgroup analysis stratified by publication date demonstrated significant effectiveness of studies published after 2010 on glycemic control in children and adolescents with T1DM compared with those published before 2010. Moreover, a decrease in heterogeneity and statistically significant subgroup differences was found in the subgroup analysis based on publication date (*P*=.01), which can explain the heterogeneity in overall effect on HbA_1c_ levels.

### Duration of Telemedicine Interventions

We created 2 subgroups: interventions lasting <6 months and interventions lasting at least 6 months. The results revealed that telemedicine interventions lasting <6 months demonstrated a more significant reduction in HbA_1c_ levels (MD –0.32, 95% CI –0.48 to –0.17; *P*<.001).

### Health Care Provider of Telemedicine Interventions

Subgroup analysis based on health care provider demonstrated significant effectiveness with or without the professional diabetes care team, and similar MDs were reported between the 2 groups (with care team: MD –0.21, 95% CI –0.38 to –0.04, and *P*=.02; without care team: MD –0.23, 95% CI –0.40 to –0.06, and *P*=.01).

### Feedback Frequency of Telemedicine Interventions

Contrary to the nonsignificant overall effect of –0.01 on HbA_1c_ levels in 25% (5/20) of the studies with feedback (not reported), the overall effect in 25% (5/20) of the studies with feedback (more than once a month) was –0.23 (95% CI –0.449 to –0.002; *P*=.048), and the overall effect in 50% (10/20) of the studies with feedback (less than or equal to once a month) was –0.27 (95% CI –0.41 to –0.12; *P<*.001); the results of the study were statistically significant.

### Communication Forms Between Patients and Providers

The choice of provider-to-patient communication forms—smartphone apps (MD –0.37, 95% CI –0.53 to –0.21; *P*<.001) and web conferences (MD –0.34, 95% CI –0.65 to –0.02; *P*=.04)—significantly influenced the effect of telemedicine on HbA_1c_ levels. In addition, the choice of patient-to-provider communication in the form of smartphone apps (MD –0.37, 95% CI –0.51 to –0.22; *P*<.001), smart wearable devices (MD –0.20, 95% CI –0.37 to –0.03; *P*=.02), and websites (MD –0.53, 95% CI –0.89 to –0.18; *P*=.003) had a significant impact on the effect on HbA_1c_ levels. A statistically significant subgroup difference was found in the subgroup analysis based on patient-to-provider communication forms (*P*=.002).

### Forms of Technology

Subgroup analysis by forms of technology showed that studies using software (MD –0.23, 95% CI –0.36 to –0.11; *P*<.001) had a significant effect on glycemic control in children and adolescents with T1DM compared with studies using only hardware (MD –0.08, 95% CI –0.51 to 0.35; *P*=.71).

### Modes of Technology Use

The overall effect on HbA_1c_ levels in the 30% (6/20) of the studies with independent use of technology was –0.11 (95% CI –0.31 to 0.09; *P*=.27), whereas the overall effect on HbA_1c_ levels in the 45% (9/20) of the studies with parental assistance was –0.24 (95% CI –0.41 to –0.07; *P*<.001).

### Forms of Telemedicine Interventions

Subgroup analysis based on the form of telemedicine intervention showed that complete telemedicine interventions (MD –0.28, 95% CI –0.43 to –0.13; *P*<.001) were better than partial telemedicine interventions (MD –0.14, 95% CI –0.29 to 0.01; *P*=.06).

### Content of Telemedicine Interventions

Interventions with interactive communication and follow-up (MD –0.27, 95% CI –0.38 to –0.17; *P*<.001) and medication dose adjustment (MD –0.25, 95% CI –0.37 to –0.12; *P*<.001) were associated with a greater improvement in HbA_1c_ levels. However, interventions without a physical exercise feature also significantly influenced the effect of telemedicine on HbA_1c_ levels (MD –0.21, 95% CI –0.35 to –0.07; *P*=.004). Moreover, a decrease in heterogeneity and statistically significant subgroup differences was found in the subgroup analysis based on interactive communication and follow-up (*P*=.002), which can also explain the heterogeneity in the overall effect on HbA_1c_ levels.

### Sensitivity Analysis

Leave-one-out analysis was performed by removing each study, and there was no significant change in the effect size (Figure S2 in Multimedia Appendix 1). Accordingly, no individual study had a statistically significant effect on the overall result. However, inspection of the effect size identified one outlier study [25] with an effect size larger than that of the other studies. The exclusion of this study did not materially affect our results for the primary outcome, but it did reduce heterogeneity (*I*^2^=9%; *Q*_18_=19.87; *P*=.34; fixed-effects model) and increase the impact of telemedicine (MD –0.26, 95% CI –0.36 to –0.17; *P*<.001).

### Publication Bias

The contour funnel plot of HbA_1c_ levels was not obviously asymmetrical, consistent with publication bias (Figure S3 in Multimedia Appendix 1). We used the Egger regression test and Begg test to verify publication bias. The regression analysis bias estimate was insignificant (Egger test: bias=–1.02 and *P=*.32; Begg test: *z=*0.16 and *P=*.87).

### Meta-Regression

The results of the meta-regression are presented in Table S2 in Multimedia Appendix 1. Meta-regression analysis showed that publication date (*P=*.04) and the “Interactive follow-up” intervention characteristic (*P=*.02) were moderating factors to explain the heterogeneity in this study.

## Discussion

### Principal Findings

In this systematic review and meta-analysis of RCTs comparing telemedicine with usual care, the difference in HbA_1c_ levels was in favor of telemedicine (MD –0.22; *P*<.001). Sensitivity analysis showed low heterogeneity (*I*^2^=35%; *P*=.06) and stability of the outliers. Subgroup analyses revealed that studies published after 2010, studies with <6 months of follow-up, studies in children with T1DM, studies in the form of smartphone apps (provider to patient) and websites (patient to provider) for communication, and studies with medication dose adjustment reported significantly larger effects of telemedicine. We were delighted to find that smartphone apps may be a particularly effective way of connecting providers and patients and that telemedicine improves quality of life for children and adolescents with T1DM (SMD –0.24, 95% CI –0.45 to –0.02; *P*=.04; *I*^2^=0%). However, there was no direct evidence that telemedicine could reduce the risk of hypoglycemia and improve SMBG. Our findings may help guide future clinical decision-making about the use of telemedicine for T1DM in children and adolescents.

### Comparison With Prior Work

Our results showed that telemedicine interventions significantly reduced HbA_1c_ levels in children and adolescents with T1DM, which is similar to the results of previous meta-analyses in adults [20,55-57]. A recent study pointed out that a telemedicine intervention for HbA_1c_ in adults had a significant treatment effect [18]. In addition, Shulman et al [58] found no evidence for the effectiveness of telemedicine on HbA_1c_ levels in a 2010 meta-analysis specifically targeting T1DM in adolescents, which is consistent with the results of this study’s time-of-publication subgroup analysis. This suggested that telemedicine has evolved and improved rapidly over the past decade or so and is showing benefits for the treatment of children and adolescents with T1DM. In addition, the results of this study are contrary to the findings of the study by Lee et al [20], which did not find that telemedicine improved glycemic control in children and adolescents with T1DM by subgroup analysis.

Although improvements in the secondary outcomes of hypoglycemia risk and SMBG were not confirmed, it is encouraging to find that telemedicine improves quality of life in children and adolescents with T1DM. This is in contrast to previous studies with adolescents and children, where Shulman et al [58] did not find differences in quality of life between the telemedicine and control groups, and is also contrary to the results of previous studies [55,57] that did not restrict the type of diabetes and studies on T1DM [20] that did not restrict the population, which did not find a benefit of telemedicine in terms of quality of life.

In terms of studying the effect of follow-up time on HbA_1c_ levels, previous studies (not specifically for T1DM) [55,57,59] have shown that the effectiveness of telemedicine is higher when the intervention duration is at least 6 months. However, our findings are contrary to those presented in these studies. Our subgroup analysis showed a higher treatment effect in studies that lasted <6 months than in studies that lasted at least 6 months. This may be related to the “honeymoon” phase of T1DM. A “honeymoon” phase is a transient period of T1DM remission characterized by a significant reduction in insulin requirements and good glycemic control due to a temporary restoration of pancreatic β-cell function, which usually lasts for several months. The exact mechanisms are still uncertain, but one of the generally recognized mechanisms is that correction of “glucotoxicity” by exogenous insulin therapy leads to “β-cell rest” and β-cell recovery [60]. The concept of a “honeymoon” phase was first described by Jackson et al [61]. They observed a rapid decline in demand for exogenous insulin in children with diabetes after regular insulin treatment. In general, patients enter the “honeymoon” period approximately 3 months after starting insulin therapy, and it can last 6 to 9 months. Therefore, it is reasonable to speculate that, in T1DM studies with shorter intervention durations, patients are more likely to be influenced by the “honeymoon” period and, thus, show a better intervention effect. Future RCTs in this area should carefully consider the duration of telemedicine interventions in their design, which should be >6 months if possible, especially if it is not sufficiently known whether the enrolled group is in or has passed the “honeymoon” period. This is to minimize the effects of the intervention being influenced by the “honeymoon” period and improve the realism and reliability of the effectiveness of telemedicine interventions. In addition, this may be related to the fact that this study targeted children and adolescents with T1DM. An alternative explanation might be that patients become less responsive to monitoring prompts as the potential novelty of telemedicine interventions diminishes. This explanation is well recognized in the related area of activity tracking via smart wearable devices [62].

Our subgroup analysis results suggested differences between children and adolescents. Telemedicine interventions had a greater effect in children compared with adolescents. This contrasts with the findings of the study by Shulman et al [58], which showed no difference in HbA_1c_ levels between the adolescent and child subgroups at the end of the intervention. It may also be due to the use of different criteria for defining children in this study from those used by Shulman et al [58]. The most recent age criteria for children and adolescents used in this study limit the age of children to less than or equal to 10 years; however, based on speculation about the publication date of the study by Schulman et al [58], they may have defined the age of the children as older. Thus based on the age criteria of the present study, we anticipate that more child-related studies in the future may make this difference more apparent. By conducting subgroup analyses, we preliminarily excluded the influence of technology forms and use modes on this result. A total of 10% (2/20) of the studies were conducted on children, one using a hardware device independently [32] and the other using software with parental assistance [36]. However, we found that the studies on children were all complete telemedicine interventions. Subgroup analysis based on intervention form showed that complete telemedicine interventions were better than partial telemedicine interventions, which could explain the observed results. This finding is supported by the study by Chen et al [63], which found that a mixed complete telemedicine intervention was superior to a partial telemedicine intervention in reducing the incidence of pressure injury in patients with spinal cord injury. Another plausible explanation is that children’s blood glucose is more prone to fluctuations and a higher incidence of hypoglycemia compared to that of adolescents, which may lead to an exaggerated intervention effect. Although HbA_1c_ is the gold standard for long-term glycemic control, the use of HbA_1c_ alone to assess glycemic management in children can be misleading due to the magnitude of blood glucose fluctuations [64], and the pursuit of HbA_1c_ compliance can be accompanied by an increase in the frequency of hypoglycemia [65,66]. Hypoglycemia in children is a metabolic-endocrine emergency due to the potential for brain injury; permanent neurological sequelae; and, in rare cases, death [67]. Therefore, when assessing glycemic control in children, special attention should be paid to the incidence of hypoglycemia. We also found that telemedicine interventions with medication dose adjustment reported significant treatment effects in improving glycemic control in children and adolescents, consistent with the results of a study [55] on the effects of telemedicine on HbA_1c_ levels in patients with diabetes. Consequently, future well-designed studies should consider further enhancing insulin adjustment and monitoring in the intervention.

On the basis of the subgroup analysis by communication form, our results suggested that smartphone apps may be a very effective vehicle for linking intervention providers and patients, which can provide an intelligent management pathway for blood glucose in children and adolescents with T1DM. Nkhoma et al [68] also supported that smartphone apps improved glycemic control better than other tools. Moreover, the smartphone app studies included in this review (5/20, 25%) all evaluated the safety of apps and reported the incidence of adverse events such as hypoglycemia and diabetic ketoacidosis. Overall, smartphone apps are safe and do not increase the number of episodes of hypoglycemia [69]. Future studies could conduct an in-depth analysis of various types of smartphone apps in terms of core functionality (eg, health monitoring, smart health interventions and guidance, community interactions, and professional support), interface design and interaction experience, and dynamic sensing and self-adaptation (eg, automatically recommending personalized health plans based on the user’s basic information, such as age, gender, and body weight) to further improve the telemedicine intervention’s usability and effectiveness. This will enable children or adolescents with T1DM to benefit more from telemedicine.

Concerning cost-effectiveness, evidence is still lacking. Few studies included in this meta-analysis (1/20, 5%) discussed cost considerations, which is a common issue faced by telemedicine intervention studies. However, there are specific telemedicine cost analysis studies that may provide assistance with cost considerations. In a recently published study on the cost-effectiveness of telemedicine interventions, smartphone app, SMS text messaging, and website interventions were confirmed to be cost-effective without substantial differences among the different delivery modes [70]. A study by Elliott et al [71] showed that smart wearable devices increase short-term costs but their HbA_1c_-lowering benefits will provide sufficient long-term health benefits and cost savings to justify the costs as long as the effects last into the medium term. The implementation of telemedicine services continues to be limited by cost and reimbursement barriers; future studies should increase transparency and conduct rigorous and in-depth cost-effectiveness analyses of the various types of telemedicine strategies to support T1DM management.

### Practice, Policy, and Future Study

Our findings have potential ramifications for practice and policy. First, among studies evaluating the use of telemedicine interventions to improve care for children and adolescents with T1DM, we found that all (20/20, 100%) focused on HbA_1c_, with only a small proportion of studies (9/20, 45%) reporting other outcomes such as quality of life and incidence of hypoglycemia. This prevents policy makers from considering the impact of interventions on outcomes other than HbA_1c_ when developing and implementing telemedicine interventions for this population. This situation may result in the health care system failing to respond to the needs of children and adolescents with T1DM and creates difficulties in tailoring telemedicine interventions to this population [72]. Focusing only on HbA_1c_ may, in turn, compromise the continuity of managed care for patients with T1DM. Therefore, we suggest that future studies add the assessment of other important outcomes such as quality of life, incidence of hypoglycemia, SMBG, and cost-effectiveness.

However, the importance of HbA_1c_ is undisputed, with findings published by the UK Prospective Diabetes Study as early as 2000 showing that a 1% reduction in mean HbA_1c_ levels was associated with a 21% reduction in diabetes-related deaths, a 14% reduction in the risk of myocardial infarction, and a 37% reduction in microvascular complications in patients with type 2 diabetes mellitus [73]. Results of a recent cross-sectional study of 156,090 children and adolescents with T1DM showed that the probability of diabetic retinopathy increased with increasing HbA_1c_ levels (adjusted odds ratio per 1 mmol/mol increase in HbA_1c_ levels 1.03, 95% CI 1.03-1.03; *P*<.001) [74]. Therefore, if telemedicine could be implemented in all children and adolescents with T1DM, it would help reduce the risk of macrovascular and microvascular complications, improve glycemic control, and enhance quality of life.

In light of the aforementioned, our findings suggest a promising application of telemedicine in the management of the disease in children and adolescents with T1DM, especially after several decades of development, during which telemedicine has shown many benefits for children and adolescents with T1DM. Future studies should carefully consider the various forms of interventions as well as the age group of the target population when tailoring telemedicine interventions for T1DM in adolescents and children, particularly with regard to the need for self-monitoring and recognition of hypoglycemia. Although the results of this study suggest that smartphone apps may be the best way to improve patients’ glycemic control, they may not be applicable to children aged <10 years. Taking China as an example, in addition to Chinese education policy discouraging the use of electronic devices in schools to minimize disruption and promote traditional teaching methods, children’s weaker self-control and potential addiction to gaming and entertainment, difficulties in parental supervision, and adverse effects on children’s face-to-face interactions and social skill development are important factors that make it difficult to apply this form of telemedicine.

Finally, this study also identified the lowest threshold of intervention duration intervals that may be able to safeguard the effectiveness of telemedicine interventions in children and adolescents with T1DM, making it necessary to conduct further studies with longer durations and larger cohort sizes in the future to determine the optimal intervals of intervention duration. Although this may be difficult; patients’ ability to improve their self-management of glycemia through telemedicine is a gradual process involving multiple factors, including patients’ learning ability, adaptability, acceptance of the technology, and the level of support from the health care team; and the time to achieve independent glycemic management may vary due to individual differences, the conduct of studies of longer durations is still very much appreciated.

### Strengths and Limitations

This systematic review and meta-analysis has several strengths. To our knowledge, this is the first meta-analysis on telemedicine aimed at improving HbA_1c_ levels in children and adolescents. The substantial number of included RCTs and participants provided strong evidence for the clinical application of telemedicine for improving glycemic control in children and adolescents with T1DM. Second, we performed a relatively comprehensive subgroup analysis and confirmed that telemedicine may have the opposite effect in children and adolescents than in adults in terms of intervention duration. In addition, we undertook a comprehensive search of multiple databases and strictly adhered to methodological tools to report our research. Finally, we performed a leave-one-out sensitivity analysis, which allowed us to assess whether high-risk studies influenced the final results; however, excluding the high-risk study did not change the final results.

We also acknowledge that this meta-analysis has several limitations, mainly statistical assumptions such as deriving the mean and SD from the sample size, baseline, end point, and median, although these assumptions were robust in several sensitivity analyses. Second, data extraction could have included more baseline data from the study, such as medication use since diagnosis (total daily insulin dose, number of insulin injections per day, and insulin pump use), ethnicity, and nationality. Third, there was a certain degree of heterogeneity in the different types of telemedicine interventions. However, subgroup analysis should overcome this flaw. Fourth, only RCTs were included in this research; observational studies may yield pertinent insights for the correlation between telemedicine and HbA_1c_ levels. Fifth, most RCTs (15/20, 75%) did not explicitly report blinding or allocation concealment procedures because of intervention characteristic limitations, which would lead to performance and detection biases. Sixth, the precision of some secondary outcomes was relatively low because of the small number of relevant trials. More RCTs of high quality and with large sample sizes are needed for further validation. Finally, only articles published in English were reviewed, which would lead to potential selection bias, and therefore, the results’ generalizability may be limited.

### Conclusions

Our systematic review and meta-analysis has shown that telemedicine is an efficacious and safe intervention approach. It can reduce HbA_1c_ levels and improve quality of life in children and adolescents with T1DM. Complete telemedicine interventions are better than partial telemedicine interventions. However, in accordance with the idea of providing health care from a distance, telemedicine should not be regarded as a uniform approach to medication or as an alternative to usual care but rather as a useful supplement to usual care to control HbA_1c_ levels and a potentially cost-effective mode. Given the potential benefits of telemedicine, such as greater access for remote populations or people with ambulatory restrictions, these findings may encourage further implementation of eHealth strategies for T1DM management, particularly as part of multifaceted interventions for integrated care of chronic diseases. The aforementioned conclusions need to be further verified in future studies. Meanwhile, researchers should develop higher-quality RCTs using large samples that focus on hard clinical outcomes, cost-effectiveness, and quality of life.

## Appendix

Multimedia Appendix 1 Search strategy, funnel plot for primary outcome, forest plots of the subgroups and the secondary outcomes, summary of meta-regression results, and sensitivity analysis.

Multimedia Appendix 2 PRISMA (Preferred Reporting Items for Systematic Reviews and Meta-Analyses) 2020 checklist.

## Abbreviations

DQOLY: Diabetes Quality of Life for Youth
HbA_1c_: glycated hemoglobin
MD: mean difference
N-QOL: non–youth-specific quality of life
PRISMA: Preferred Reporting Items for Systematic Reviews and Meta-Analyses
RCT: randomized controlled trial
SMBG: self-monitoring of blood glucose
SMD: standardized mean difference
T1DM: type 1 diabetes mellitus
