# Supplementary material for: Telemedicine in Improving Glycemic Control Among Children and Adolescents With Type 1 Diabetes Mellitus: Systematic Review and Meta-Analysis
Source: J Med Internet Res. 2024 Jul 9;26:e51538. doi: 10.2196/51538 (PMC11267117; doi:10.2196/51538)
Supplement: Multimedia Appendix 1 [file jmir_v26i1e51538_app1.docx]

**Telemedicine in improving glycemic control among children and adolescents with type 1**

**diabetes mellitus: A systematic review and meta-analysis**

**Online Supplementary material**

**Search strategy**

**Pubmed, CINHAL complete**

#1

(((((((((((((((((((((((((((Diabetes Mellitus, Type 1[MeSH Terms]) OR (Diabetes Mellitus, Insulin-Dependent[Title/Abstract])) OR (Diabetes Mellitus, Insulin Dependent[Title/Abstract])) OR (Insulin-Dependent Diabetes Mellitus[Title/Abstract])) OR (Diabetes Mellitus, Juvenile-Onset[Title/Abstract])) OR (Diabetes Mellitus, Juvenile Onset[Title/Abstract])) OR (Juvenile-Onset Diabetes Mellitus[Title/Abstract])) OR (IDDM[Title/Abstract])) OR (Juvenile-Onset Diabetes[Title/Abstract])) OR (Diabetes, Juvenile-Onset[Title/Abstract])) OR (Juvenile Onset Diabetes[Title/Abstract])) OR (Diabetes Mellitus, Sudden-Onset[Title/Abstract])) OR (Diabetes Mellitus, Sudden Onset[Title/Abstract])) OR (Sudden-Onset Diabetes Mellitus[Title/Abstract])) OR (Type 1 Diabetes Mellitus[Title/Abstract])) OR (Diabetes Mellitus, Insulin-Dependent, 1[Title/Abstract])) OR (Insulin-Dependent Diabetes Mellitus 1[Title/Abstract])) OR (Insulin Dependent Diabetes Mellitus 1[Title/Abstract])) OR (Type 1 Diabetes[Title/Abstract])) OR (Diabetes, Type 1[Title/Abstract])) OR (Diabetes Mellitus, Type I[Title/Abstract])) OR (Diabetes, Autoimmune[Title/Abstract])) OR (Autoimmune Diabetes[Title/Abstract])) OR (Diabetes Mellitus, Brittle[Title/Abstract])) OR (Brittle Diabetes Mellitus[Title/Abstract])) OR (Diabetes Mellitus, Ketosis-Prone[Title/Abstract])) OR (Diabetes Mellitus, Ketosis Prone[Title/Abstract])) OR (Ketosis-Prone Diabetes Mellitus[Title/Abstract])

#2

((((((((((((((((((((((((((((((((((((((((((((((((((((((((((((((((((((((((Text Messaging[MeSH Terms]) OR (Internet-Based Intervention[MeSH Terms])) OR (Telemedicine[MeSH Terms])) OR (Telemetry[MeSH Terms])) OR (Telenursing[MeSH Terms])) OR (Smartphone[MeSH Terms])) OR (Computers[MeSH Terms])) OR (Messaging, Text[Title/Abstract])) OR (Texting[Title/Abstract])) OR (Textings[Title/Abstract])) OR (Short Message Service[Title/Abstract])) OR (Text Messages[Title/Abstract])) OR (Message, Text[Title/Abstract])) OR (Messages, Text[Title/Abstract])) OR (Text Message[Title/Abstract])) OR (Internet Based Intervention[Title/Abstract])) OR (Internet-Based Interventions[Title/Abstract])) OR (Intervention, Internet-Based[Title/Abstract])) OR (Interventions, Internet-Based[Title/Abstract])) OR (Web-based Intervention[Title/Abstract])) OR (Intervention, Web-based[Title/Abstract])) OR (Interventions, Web-based[Title/Abstract])) OR (Web based Intervention[Title/Abstract])) OR (Web-based Interventions[Title/Abstract])) OR (Online Intervention[Title/Abstract])) OR (Intervention, Online[Title/Abstract])) OR (Interventions, Online[Title/Abstract])) OR (Online Interventions[Title/Abstract])) OR (Internet Intervention[Title/Abstract])) OR (Internet Interventions[Title/Abstract])) OR (Intervention, Internet[Title/Abstract])) OR (Interventions, Internet[Title/Abstract])) OR (Tele-Referral[Title/Abstract])) OR (Tele Referral[Title/Abstract])) OR (Tele-Referrals[Title/Abstract])) OR (Virtual Medicine[Title/Abstract])) OR (Medicine, Virtual[Title/Abstract])) OR (Tele-Intensive Care[Title/Abstract])) OR (Tele Intensive Care[Title/Abstract])) OR (Tele-ICU[Title/Abstract])) OR (Tele ICU[Title/Abstract])) OR (Mobile Health[Title/Abstract])) OR (Health, Mobile[Title/Abstract])) OR (mHealth[Title/Abstract])) OR (Telehealth[Title/Abstract])) OR (eHealth[Title/Abstract])) OR (Telemetries[Title/Abstract])) OR (Smartphones[Title/Abstract])) OR (Smart Phones[Title/Abstract])) OR (Smart Phone[Title/Abstract])) OR (Phones, Smart[Title/Abstract]))) OR (Computer[Title/Abstract])) OR (Calculators, Programmable[Title/Abstract])) OR (Calculator, Programmable[Title/Abstract])) OR (Programmable Calculator[Title/Abstract])) OR (Programmable Calculators[Title/Abstract])) OR (Hardware, Computer[Title/Abstract])) OR (Computer Hardware[Title/Abstract])) OR (Computers, Digital[Title/Abstract])) OR (Computer, Digital[Title/Abstract])) OR (Digital Computer[Title/Abstract])) OR (Digital Computers[Title/Abstract])

#3

((((((((((((((((((child[MeSH Terms]) OR (Adolescent[MeSH Terms])) OR (children[Title/Abstract])) OR (Adolescents[Title/Abstract])) OR (Adolescence[Title/Abstract])) OR (Teens[Title/Abstract])) OR (Teen[Title/Abstract])) OR (Teenagers[Title/Abstract])) OR (Teenager[Title/Abstract])) OR (Youth[Title/Abstract])) OR (Youths[Title/Abstract])) OR (Adolescents, Female[Title/Abstract])) OR (Adolescent, Female[Title/Abstract])) OR (Female Adolescent[Title/Abstract])) OR (Female Adolescents[Title/Abstract])) OR (Adolescents, Male[Title/Abstract])) OR (Adolescent, Male[Title/Abstract])) OR (Male Adolescent[Title/Abstract])) OR (Male Adolescents[Title/Abstract])

#4

((((((((((((((((((((((((((((((Self-Management[MeSH Terms]) OR (Blood Glucose[MeSH Terms])) OR (Blood Glucose Self-Monitoring[MeSH Terms])) OR (Glycemic Control[MeSH Terms])) OR (Self Management[Title/Abstract])) OR (Management, Self[Title/Abstract])) OR (Blood Sugar[Title/Abstract])) OR (Sugar, Blood[Title/Abstract])) OR (Glucose, Blood[Title/Abstract])) OR (Blood Glucose Self Monitoring[Title/Abstract])) OR (Glucose, Blood, Self-Monitoring[Title/Abstract])) OR (Self-Monitoring, Blood Glucose[Title/Abstract])) OR (Blood Glucose Self-Monitorings[Title/Abstract])) OR (Glucose Self-Monitoring, Blood[Title/Abstract])) OR (Glucose Self-Monitorings, Blood[Title/Abstract])) OR (Self Monitoring, Blood Glucose[Title/Abstract])) OR (Self-Monitorings, Blood Glucose[Title/Abstract])) OR (Monitoring, Home Blood Glucose[Title/Abstract])) OR (Blood Sugar Self-Monitoring[Title/Abstract])) OR (Blood Sugar Self Monitoring[Title/Abstract])) OR (Blood Sugar Self-Monitorings[Title/Abstract])) OR (Self-Monitoring, Blood Sugar[Title/Abstract])) OR (Self-Monitorings, Blood Sugar[Title/Abstract])) OR (Sugar Self-Monitoring, Blood[Title/Abstract])) OR (Sugar Self-Monitorings, Blood[Title/Abstract])) OR (Home Blood Glucose Monitoring[Title/Abstract])) OR (Glucose, Blood, Self Monitoring[Title/Abstract])) OR (Control, Glycemic[Title/Abstract])) OR (Blood Glucose Control[Title/Abstract])) OR (Control, Blood Glucose[Title/Abstract])) OR (Glucose Control, Blood[Title/Abstract])

#5

#1 AND #2 AND #3 AND #4 Filter: Randomized Controlled Trail

**Cochrane**

ID Search Hits

#1 MeSH descriptor: [Diabetes Mellitus, Type 1] explode all trees **6729**

#2 (Diabetes Mellitus, Insulin-Dependent):ti,ab,kw OR (Diabetes Mellitus, Insulin Dependent):ti,ab.kw **26454**

#3 (Insulin-Dependent Diabetes Mellitus):ti,ab,kw OR (Diabetes Mellitus, Juvenile-Onset):ti,

ab,kw OR (Diabetes Mellitus, Juvenile Onset):ti,ab,kw OR (Juvenile-Onset, Diabetes Mellitus):ti,ab,kw OR (IDDM):ti,ab,kw OR (Juvenile-Onset, Diabetes):ti,ab,kw OR (Diabetes, Juvenile-Onset):ti,ab,kw OR (Juvenile Onset Diabetes):ti,ab,kw OR (Diabetes Mellitus, Sudden-Onset):ti,ab,kw OR (Diabetes Mellitus, Sudden Onset):ti,ab,kw OR (Sudden-Onset Diabetes Mellitus):ti,ab,kw OR (Type 1 Diabetes Mellitus):ti,ab,kw OR (Diabetes Mellitus, Insulin-Dependent, 1):ti,ab,kw OR (Insulin-Dependent Diabetes Mellitus 1):ti,ab,kw OR (Insulin Dependent Diabetes Mellitus 1):ti,ab,kw OR (Type 1 Diabetes):ti,ab,kw OR (Diabetes, Type 1):ti,ab,kw OR (Diabetes Mellitus, Type 1):ti,ab,kw OR (Diabetes, Autoimmune):ti,ab,kw OR (Autoimmune Diabetes):ti,ab,kw OR (Diabetes Mellitus, Brittle):ti,ab,kw OR (Brittle Diabetes Mellitus):ti,ab,kw OR (Diabetes Mellitus, Ketosis-Prone):ti,ab,kw OR (Diabetes Mellitus, Ketosis Prone):ti,ab,kw OR (Ketosis-Prone, Diabetes Mellitus):ti,ab,kw **52394**

#4 #1 OR #2 OR #3 **52630**

#5 MeSH descriptor: [Child] explode all trees **77487**

#6 (children):ti,ab,kw **174134**

#7 MeSH descriptor: [Adolescent] explode all trees **125167**

#8 (Adolescence):ti,ab,kw OR (Teens):ti,ab,kw OR (Teen):ti,ab,kw OR (Teenagers):ti,ab,kw OR (Teenager):ti,ab,kw OR (Youth):ti,ab,kw OR (Youths):ti,ab,kw OR (Adolescents, Female):ti,ab,kw OR (Adolescent, Female):ti,ab,kw OR (Female Adolescent):ti,ab,kw OR (Female Adolescents):ti,ab,kw OR (Adolescents, Male):ti,ab,kw OR (Adolescent, Male):ti,ab,kw OR (Male Adolescent):ti,ab,kw OR (Male Adolescents):ti,ab,kw **139752**

#9 #5 OR #6 OR #7 OR #8 **273013**

#10 #4 AND #9 **5676**

#11 MeSH descriptor: [Text Messaging] explode all trees **1474**

#12 (Messaging, Text):ti,ab,kw OR (Texting):ti,ab,kw OR (Textings):ti,ab,kw OR (Short Message Service):ti,ab,kw OR (Text Messages):ti,ab,kw OR (Message, Text):ti,ab,kw OR (Messages, Text):ti,ab,kw OR (Text Message):ti,ab,kw **6345**

#13 MeSH descriptor: [Internet-Based Intervention] explode all trees **539**

#14 (Internet Based Intervention):ti,ab,kw OR (Internet-Based Interventions):ti,ab,kw OR (Intervention, Internet-Based):ti,ab,kw OR (Interventions, Internet-Based):ti,ab,kw OR (Web-based Intervention):ti,ab,kw OR (Intervention, Web-based):ti,ab,kw OR (Interventions, Web-based):ti,ab,kw OR (Web based Intervention):ti,ab,kw OR (Web-based Interventions):ti,ab,kw OR (Online Intervention):ti,ab,kw OR (Intervention, Online):ti,ab,kw OR (Interventions, Online):ti,ab,kw OR (Online Interventions):ti,ab,kw OR (Internet Intervention):ti,ab,kw OR (Internet Interventions):ti,ab,kw OR

(Intervention, Internet):ti,ab,kw OR (Interventions, Internet):ti,ab,kw **23848**

#15 MeSH descriptor: [Telemedicine] explode all trees **4182**

#16 (Tele-Referral):ti,ab,kw OR (Tele Referral):ti,ab,kw OR (Tele-Referrals):ti,ab,kw OR (Virtual Medicine):ti,ab,kw OR (Medicine, Virtual):ti,ab,kw OR (Tele-Intensive Care):ti,ab,kw OR (Tele Intensive Care):ti,ab,kw OR (Tele-ICU):ti,ab,kw OR (Tele ICU):ti,ab,kw OR (Mobile Health):ti,ab,kw OR (Health, Mobile):ti,ab,kw OR (mHealth):ti,ab,kw OR (Telehealth):ti,ab,kw OR (eHealth):ti,ab,kw **12823**

#17 MeSH descriptor: [Telemetry] explode all trees **377**

#18 (Telemetries):ti,ab,kw  **0**

#19 MeSH descriptor: [Telenursing] explode all trees **45**

#20 MeSH descriptor: [Smartphone] explode all trees **999**

#21 (Smartphones):ti,ab,kw OR (Smart Phones):ti,ab,kw OR (Smart Phone):ti,ab,kw OR (Phones, Smart):ti,ab,kw **2389**

#22 MeSH descriptor: [Computers] explode all trees **2606**

#23 (Computer):ti,ab,kw OR (Calculators, Programmable):ti,ab,kw OR (Calculator, Programmable):ti,ab,kw OR (Programmable Calculator):ti,ab,kw OR (Programmable Calculators):ti,ab,kw OR (Hardware, Computer):ti,ab,kw OR (Computer Hardware):ti,ab,kw OR (Computers, Digital):ti,ab,kw OR (Computer, Digital):ti,ab,kw OR (Digital Computer):ti,ab,kw OR (Digital Computers):ti,ab,kw **49524**

#24 #11 OR #12 OR #13 OR #14 OR #15 OR #16 OR #17 OR #18 OR #19 OR #20 OR #21 OR #22 OR #23 **86669**

#25 #10 AND #24 **560**

#26 MeSH descriptor: [Self-Management] explode all trees **979**

#27 (Self Management):ti,ab,kw OR (Management, Self):ti,ab,kw **24720**

#28 MeSH descriptor: [Blood Glucose] explode all trees **19244**

#29 (Blood Sugar):ti,ab,kw OR (Sugar, Blood):ti,ab,kw OR (Glucose, Blood):ti,ab,kw **63340**

#30 MeSH descriptor: [Blood Glucose Self-Monitoring] explode all trees **1227**

#31 (Blood Glucose Self Monitoring):ti,ab,kw OR (Glucose, Blood, Self-Monitoring):ti,ab,kw OR (Self-Monitoring, Blood Glucose):ti,ab,kw OR (Blood Glucose Self-Monitorings):ti,ab,kw OR (Glucose Self-Monitoring, Blood):ti,ab,kw OR (Glucose Self-Monitorings, Blood):ti,ab,kw OR (Self Monitoring, Blood Glucose):ti,ab,kw OR (Self-Monitorings, Blood Glucose):ti,ab,kw OR (Monitoring, Home Blood Glucose):ti,ab,kw OR (Blood Sugar Self-Monitoring):ti,ab,kw OR (Blood Sugar Self Monitoring):ti,ab,kw OR (Blood Sugar Self-Monitorings):ti,ab,kw OR (Self-Monitoring, Blood Sugar):ti,ab,kw OR (Self-Monitorings, Blood Sugar):ti,ab,kw OR (Sugar Self-Monitoring, Blood):ti,ab,kw OR (Sugar Self-Monitorings, Blood):ti,ab,kw OR (Home Blood Glucose Monitoring):ti,ab,kw OR (Glucose, Blood, Self Monitoring):ti,ab,kw **3472**

#32 MeSH descriptor: [Glycemic Control] explode all trees **1591**

#33 (Control, Glycemic):ti,ab,kw OR (Blood Glucose Control):ti,ab,kw OR (Control, Blood Glucose):ti,ab,kw OR (Glucose Control, Blood):ti,ab,kw **37061**

#34 #26 OR #27 OR #28 OR #29 OR #30 OR #31 OR #32 OR #33 **92664**

#35 #25 AND #34 **364**

#36 MeSH descriptor: [Randomized Controlled Trail] explode all trees **25743**

#37 (RCT):ti,ab,kw OR (randomized controlled trail):ti,ab,kw OR (randomized controlled):ti,ab,kw **787190**

#38 #36 OR #37 **787190**

#39 #38 AND #35 **249**

**Embase**

No. Query

#1 'insulin dependent diabetes mellitus'/exp **142573**

#2 'diabetes mellitus, insulin-dependent':ab,ti OR 'diabetes mellitus, insulin dependent':ab,ti OR 'insulin-dependent diabetes mellitus':ab,ti OR 'diabetes mellitus, juvenile-onset':ab,ti OR 'diabetes mellitus, juvenile onset':ab,ti OR 'juvenile-onset diabetes mellitus':ab,ti OR 'iddm':ab,ti OR 'juvenile-onset diabetes':ab,ti OR 'diabetes, juvenile-onset':ab,ti OR 'juvenile onset diabetes':ab,ti OR 'diabetes mellitus, sudden-onset':ab,ti OR 'diabetes mellitus, sudden onset':ab,ti OR 'sudden-onset diabetes mellitus':ab,ti OR 'type 1 diabetes mellitus':ab,ti OR 'diabetes mellitus, insulin-dependent, 1':ab,ti OR 'insulin-dependent diabetes mellitus 1':ab,ti OR 'insulin dependent diabetes mellitus 1':ab,ti OR 'type 1 diabetes':ab,ti OR 'diabetes, type 1':ab,ti OR 'diabetes mellitus, type i':ab,ti OR 'diabetes, autoimmune':ab,ti OR 'autoimmune diabetes':ab,ti OR 'diabetes mellitus, brittle':ab,ti OR 'brittle diabetes mellitus':ab,ti OR 'diabetes mellitus, ketosis-prone':ab,ti OR 'diabetes mellitus, ketosis prone':ab,ti OR 'ketosis-prone diabetes mellitus':ab,ti **102974**

#3 'child'/exp **3321568**

#4 'children':ab,ti **1646477**

#5 'adolescent'/exp **1897413**

#6 'adolescence':ab,ti OR 'teens':ab,ti OR 'teen':ab,ti OR 'teenagers':ab,ti OR 'teenager':ab,ti OR 'youth':ab,ti OR 'youths':ab,ti OR 'adolescents, female':ab,ti OR 'adolescent, female':ab,ti OR 'female adolescent':ab,ti OR 'female adolescents':ab,ti OR 'adolescents, male':ab,ti OR 'adolescent, male':ab,ti OR 'male adolescent':ab,ti OR 'male adolescents':ab,ti **237741**

#7 'text messaging'/exp **7386**

#8 'text messaging':ab,ti OR 'messaging, text':ab,ti OR 'texting':ab,ti OR 'textings':ab,ti OR 'short message service':ab,ti OR 'text messages':ab,ti OR 'message, text':ab,ti OR 'messages, text':ab,ti OR 'text message':ab,ti **9613**

#9 'web-based intervention'/exp **2498**

#10 'internet-based intervention':ab,ti OR 'internet based intervention':ab,ti OR 'internet-based interventions':ab,ti OR 'intervention, internet-based':ab,ti OR 'interventions, internet-based':ab,ti OR 'web-based intervention':ab,ti OR 'intervention, web-based':ab,ti OR 'interventions, web-based':ab,ti OR 'web based intervention':ab,ti OR 'web-based interventions':ab,ti OR 'online intervention':ab,ti OR 'intervention, online':ab,ti OR 'interventions, online':ab,ti OR 'online interventions':ab,ti OR 'internet intervention':ab,ti OR 'internet interventions':ab,ti OR 'intervention, internet':ab,ti OR 'interventions, internet':ab,ti **16023**

#11 'telemedicine'/exp **68384**

#12 'tele-referral':ab,ti OR 'tele referral':ab,ti OR 'tele-referrals':ab,ti OR 'virtual medicine':ab,ti OR 'medicine, virtual':ab,ti OR 'tele-intensive care':ab,ti OR 'tele intensive care':ab,ti OR 'tele-icu':ab,ti OR 'tele icu':ab,ti OR 'mobile health':ab,ti OR 'health, mobile':ab,ti OR 'mhealth':ab,ti OR 'telehealth':ab,ti OR 'ehealth':ab,ti **31206**

#13 'telemetry'/exp **39178**

#14 'telemetries':ab,ti **24**

#15 'telenursing'/exp **365**

#16 'smartphone'/exp **24850**

#17 'smartphones':ab,ti OR 'smart phones':ab,ti OR 'smart phone':ab,ti OR 'phones, smart':ab,ti **11432**

#18 'computer'/exp **174478**

#19 'computer':ab,ti OR 'calculators, programmable':ab,ti OR 'calculator, programmable':ab,ti OR 'programmable calculator':ab,ti OR 'programmable calculators':ab,ti OR 'hardware, computer':ab,ti OR 'computer hardware':ab,ti OR 'computers, digital':ab,ti OR 'computer, digital':ab,ti OR 'digital computer':ab,ti OR 'digital computers':ab,ti **301028**

#20 'self care'/exp **100791**

#21 'self-management':ab,ti OR 'self management':ab,ti OR 'management, self':ab,ti  **35633**

#22 'glucose blood level'/exp **325161**

#23 'blood glucose':ab,ti OR 'blood sugar':ab,ti OR 'sugar, blood':ab,ti OR 'glucose, blood':ab,ti **150646**

#24 'blood glucose monitoring'/exp  **34804**

#25 'blood glucose self-monitoring':ab,ti OR 'blood glucose self monitoring':ab,ti OR 'glucose, blood, self-monitoring':ab,ti OR 'self-monitoring, blood glucose':ab,ti OR 'blood glucose self-monitorings':ab,ti OR 'glucose self-monitoring, blood':ab,ti OR 'glucose self-monitorings, blood':ab,ti OR 'self monitoring, blood glucose':ab,ti OR 'self-monitorings, blood glucose':ab,ti OR 'monitoring, home blood glucose':ab,ti OR 'blood sugar self-monitoring':ab,ti OR 'blood sugar self monitoring':ab,ti OR 'blood sugar self-monitorings':ab,ti OR 'self-monitoring, blood sugar':ab,ti OR 'self-monitorings, blood sugar':ab,ti OR 'sugar self-monitoring, blood':ab,ti OR 'sugar self-monitorings, blood':ab,ti OR 'home blood glucose monitoring':ab,ti OR 'glucose, blood, self monitoring':ab,ti **1281**

#26 'glycemic control'/exp  **66162**

#27 'glycemic control':ab,ti OR 'control, glycemic':ab,ti OR 'blood glucose control':ab,ti OR 'control, blood glucose':ab,ti OR 'glucose control, blood':ab,ti **53309**

#28 #1 OR #2 **166592**

#29 #3 OR #4 OR #5 OR #6  **4597249**

#30 #7 OR #8 OR #9 OR #10 OR #11 OR #12 OR #13 OR #14 OR #15 OR #16 OR #17 OR

#18 OR #19 **568452**

#31 #20 OR #21 OR #22 OR #23 OR #24 OR #25 OR #26 OR #27  **525363**

#32 #28 AND #29 AND #30 AND #31  **614**

#33 'randomized controlled trial'/exp  **763641**

#34 'randomized controlled trial':ab,ti OR 'randomized controlled':ab,ti OR 'rct':ab,ti  **329148**

#35 #33 OR #34  **930507**

#36 #32 AND #35 **140**

**Web of Science( all database)**

Included: WOS: 1900 to 2023; BCI: 1994 to 2023; CSCD: 1989 to 2023; DIIDW: 1966 to 2021; FSTA: 1969 to 2023; KJD: 1980 to 2023; MEDLINE: 1950 to 2023; PPRN: 1991 to 2023; SCIELO: 2002 to 2023; ZOOREC: 1864 to 2023

1: TS=(Diabetes Mellitus, Type 1 OR Diabetes Mellitus, Insulin-Dependent OR Diabetes Mellitus, Insulin Dependent OR Insulin-Dependent Diabetes Mellitus OR Diabetes Mellitus, Juvenile-Onset OR Diabetes Mellitus, Juvenile Onset OR Juvenile-Onset Diabetes Mellitus OR IDDM OR Juvenile-Onset Diabetes OR Diabetes, Juvenile-Onset OR Juvenile Onset Diabetes OR Diabetes Mellitus, Sudden-Onset OR Diabetes Mellitus, Sudden Onset OR Sudden-Onset Diabetes Mellitus OR Type 1 Diabetes Mellitus OR Diabetes Mellitus, Insulin-Dependent, 1 OR Insulin-Dependent Diabetes Mellitus 1 OR Insulin Dependent Diabetes Mellitus 1 OR Type 1 Diabetes OR Diabetes, Type 1 OR Diabetes Mellitus, Type I OR Diabetes, Autoimmune OR Autoimmune Diabetes OR Diabetes Mellitus, Brittle OR Brittle Diabetes Mellitus OR Diabetes Mellitus, Ketosis-Prone OR Diabetes Mellitus, Ketosis Prone OR Ketosis-Prone Diabetes Mellitus) **486066**

2: TS=(child OR children OR Adolescence OR Teens OR Teen OR Teenagers OR Teenager OR Youth OR Youths OR Adolescents, Female OR Adolescent, Female OR Female Adolescent OR Female Adolescents OR Adolescents, Male OR Adolescent, Male OR Male Adolescent OR Male Adolescents) **5483635**

3: TS=(Text Messaging OR Messaging, Text OR Texting OR Textings OR Short Message Service OR Text Messages OR Message, Text OR Messages, Text OR Text Message OR Internet-Based Intervention OR Internet Based Intervention OR Internet-Based Interventions OR Intervention, Internet-Based OR Interventions, Internet-Based OR Web-based Intervention OR Intervention, Web-based OR Interventions, Web-based OR Web based Intervention OR Web-based Interventions OR Online Intervention OR Intervention, Online OR Interventions, Online OR Online Interventions OR Internet Intervention OR Internet Interventions OR Intervention, Internet OR Interventions, Internet OR Telemedicine OR Tele-Referral OR Tele Referral OR Tele-Referrals OR Virtual Medicine OR Medicine, Virtual OR Tele-Intensive Care OR Tele Intensive Care OR Tele-ICU OR Tele ICU OR Mobile Health OR Health, Mobile OR mHealth OR Telehealth OR eHealth OR Telemetry OR Telemetries OR Telenursing OR Smartphone OR Smart Phones OR Smartphones OR Smart Phone OR Phones, Smart OR Computers OR Computer OR Calculators, Programmable OR Calculator, Programmable OR Programmable Calculator OR Programmable Calculators OR Hardware, Computer OR Computer Hardware OR Computers, Digital OR Computer, Digital OR Digital Computer OR Digital Computers) **8860381**

4.TS=(Self-Management OR Self Management OR Management, Self OR Blood Glucose OR Blood Sugar OR Sugar, Blood OR Glucose, Blood OR Blood Glucose Self-Monitoring OR Blood Glucose Self Monitoring OR Glucose, Blood, Self-Monitoring OR Self-Monitoring, Blood Glucose OR Blood Glucose Self-Monitorings OR Glucose Self-Monitoring, Blood OR Glucose Self-Monitorings, Blood OR Self Monitoring, Blood Glucose OR Self-Monitorings, Blood Glucose OR Monitoring, Home Blood Glucose OR Blood Sugar Self-Monitoring OR Blood Sugar Self Monitoring OR Blood Sugar Self-Monitorings OR Self-Monitoring, Blood Sugar OR Self-Monitorings, Blood Sugar OR Sugar Self-Monitoring, Blood OR Sugar Self-Monitorings, Blood OR Home Blood Glucose Monitoring OR Glucose, Blood, Self Monitoring OR Glycemic Control OR Control, Glycemic OR Blood Glucose Control OR Control, Blood Glucose OR Glucose Control, Blood)  **1029557**

5: #4 AND #3 AND #2 AND #1 **2023**

6: TS=(randomized controlled trail OR placebo OR Randomized Controlled trail OR RCT OR randomized conrtrol) **419906**

7: #5 AND #6  **69**

**Supplementary Figure S1:** Funnel plots for primary outcome


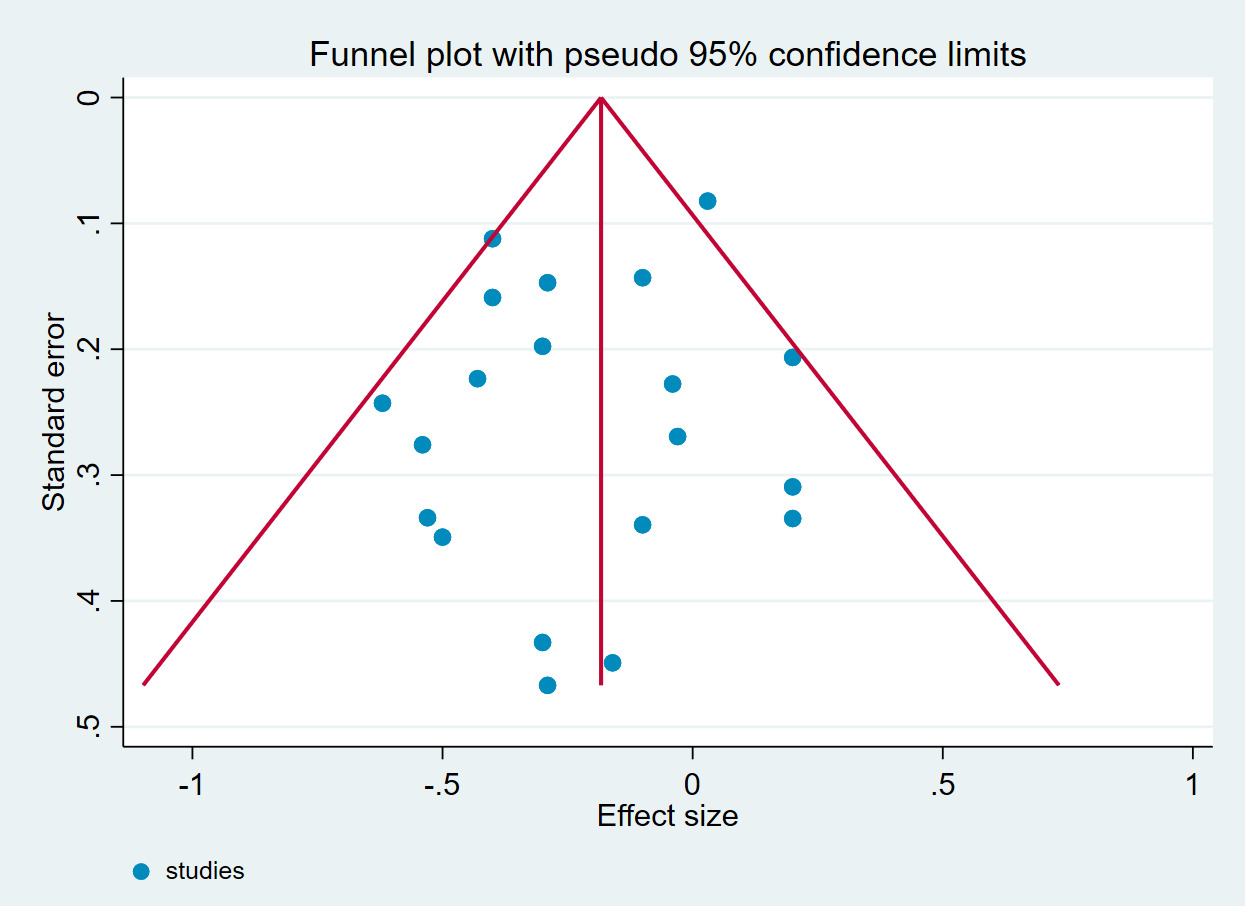


**Supplementary Figure S3:** Forest plots of the subgroups

**A. Age**

**
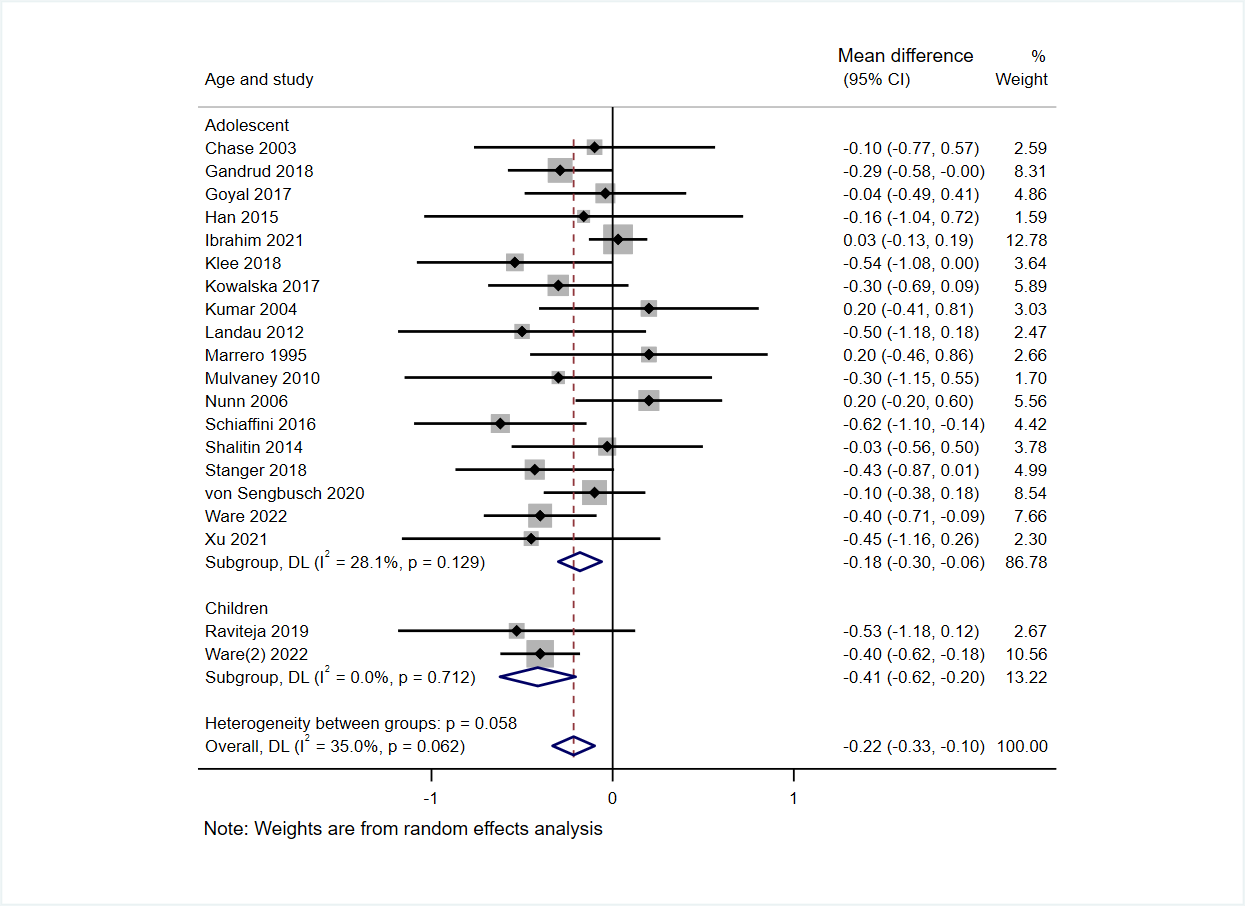
**

**B. Publication date**

**
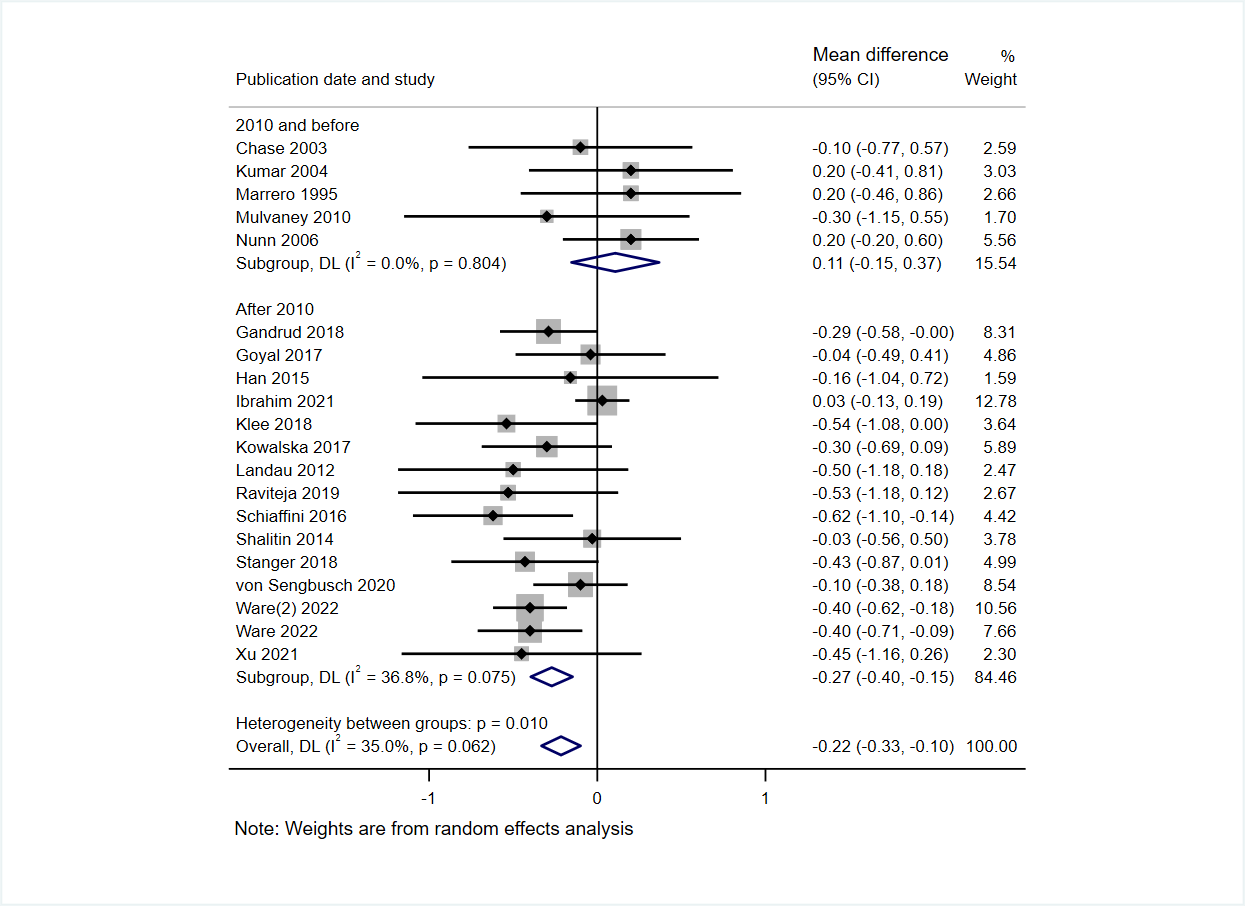
**

**C. Intervention duration**

**
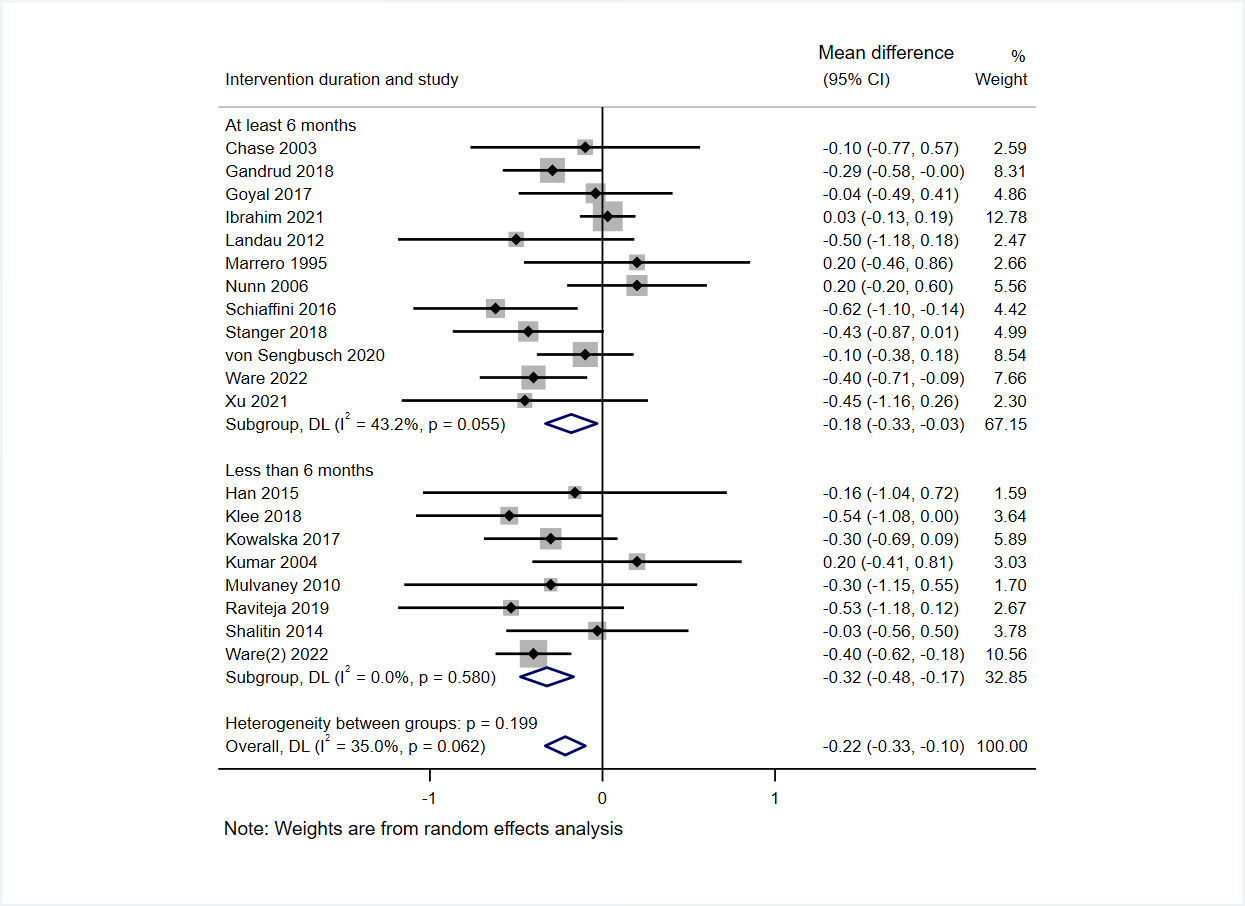
**

**D. Health care provider**

**
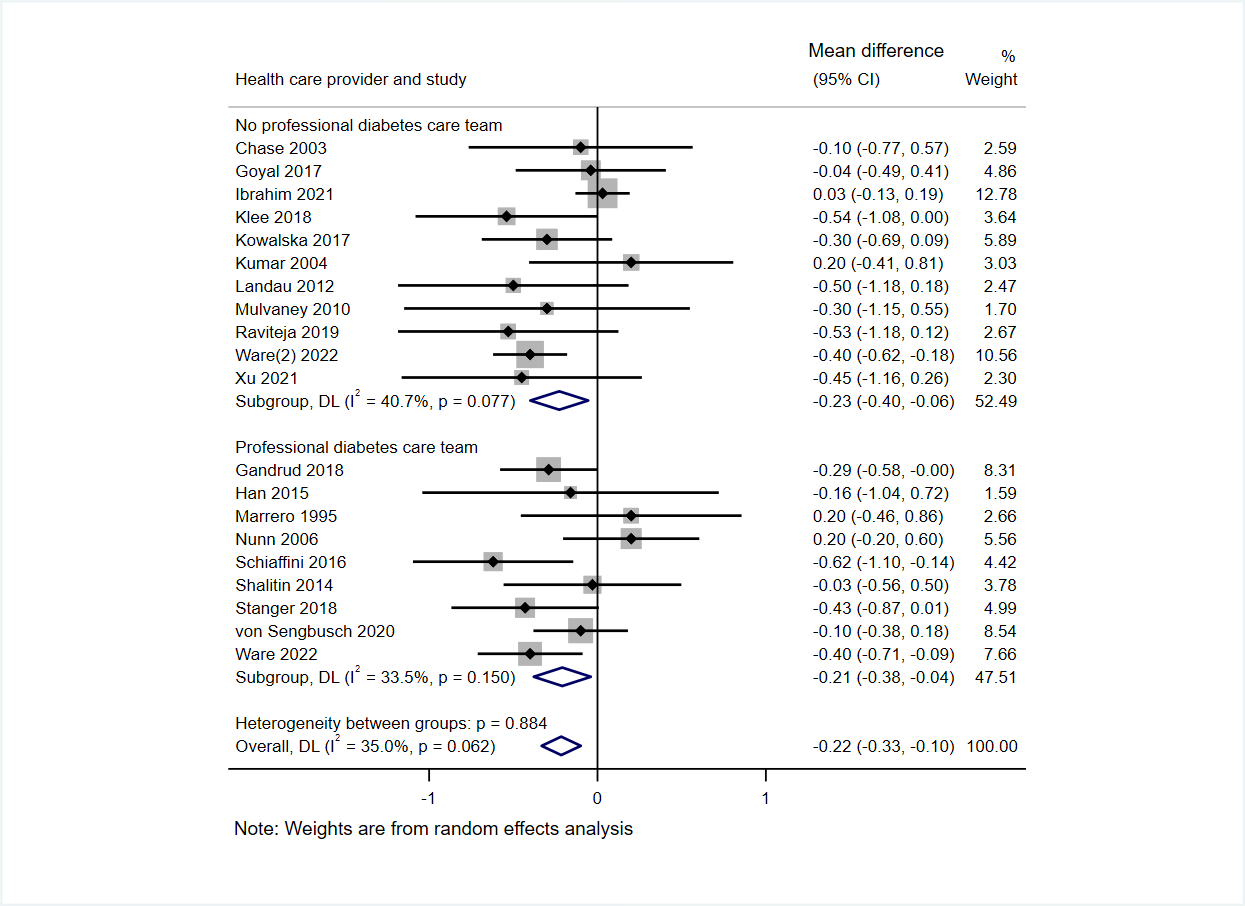
**

**E. Feedback frequency**

**
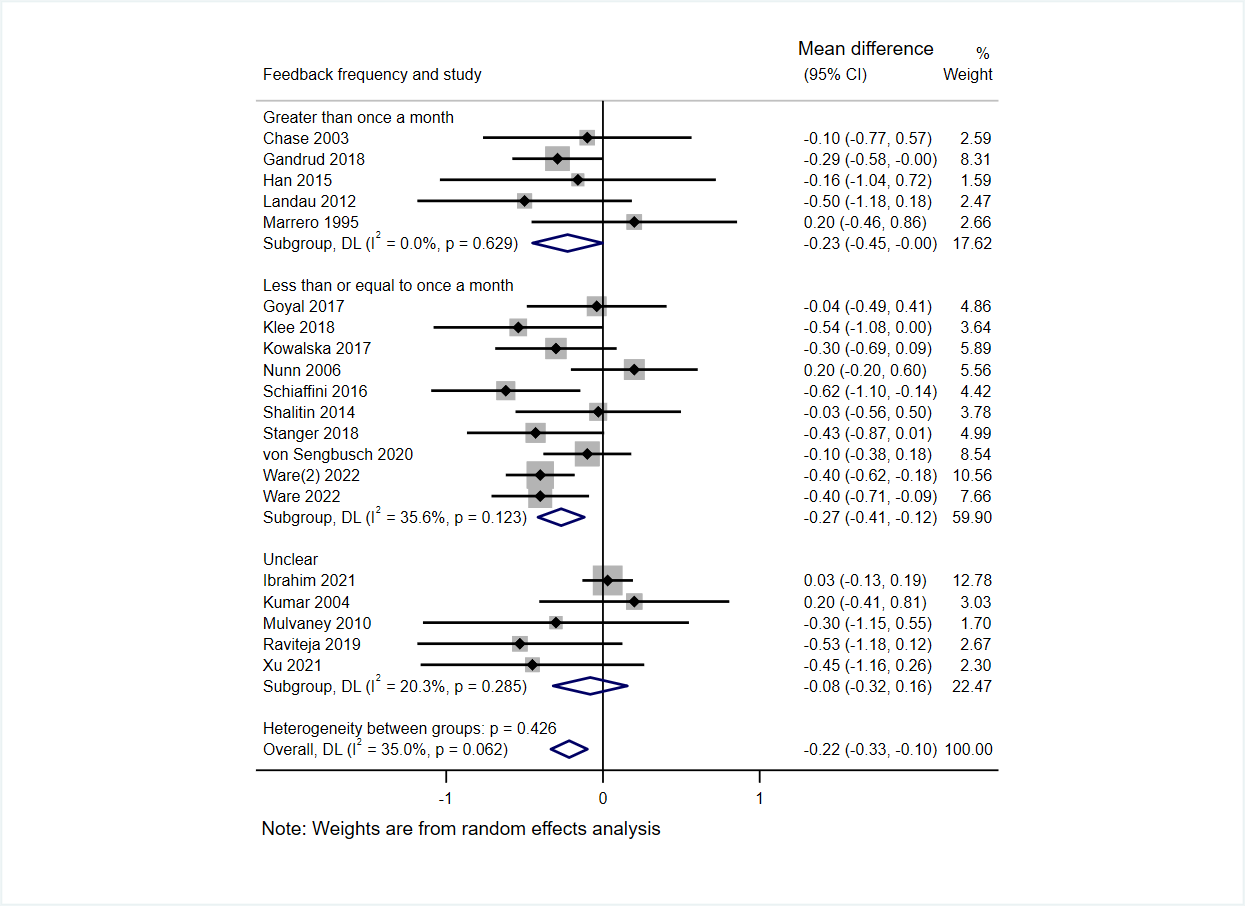
**

**F. Communication forms(provider-to-patient)**

**
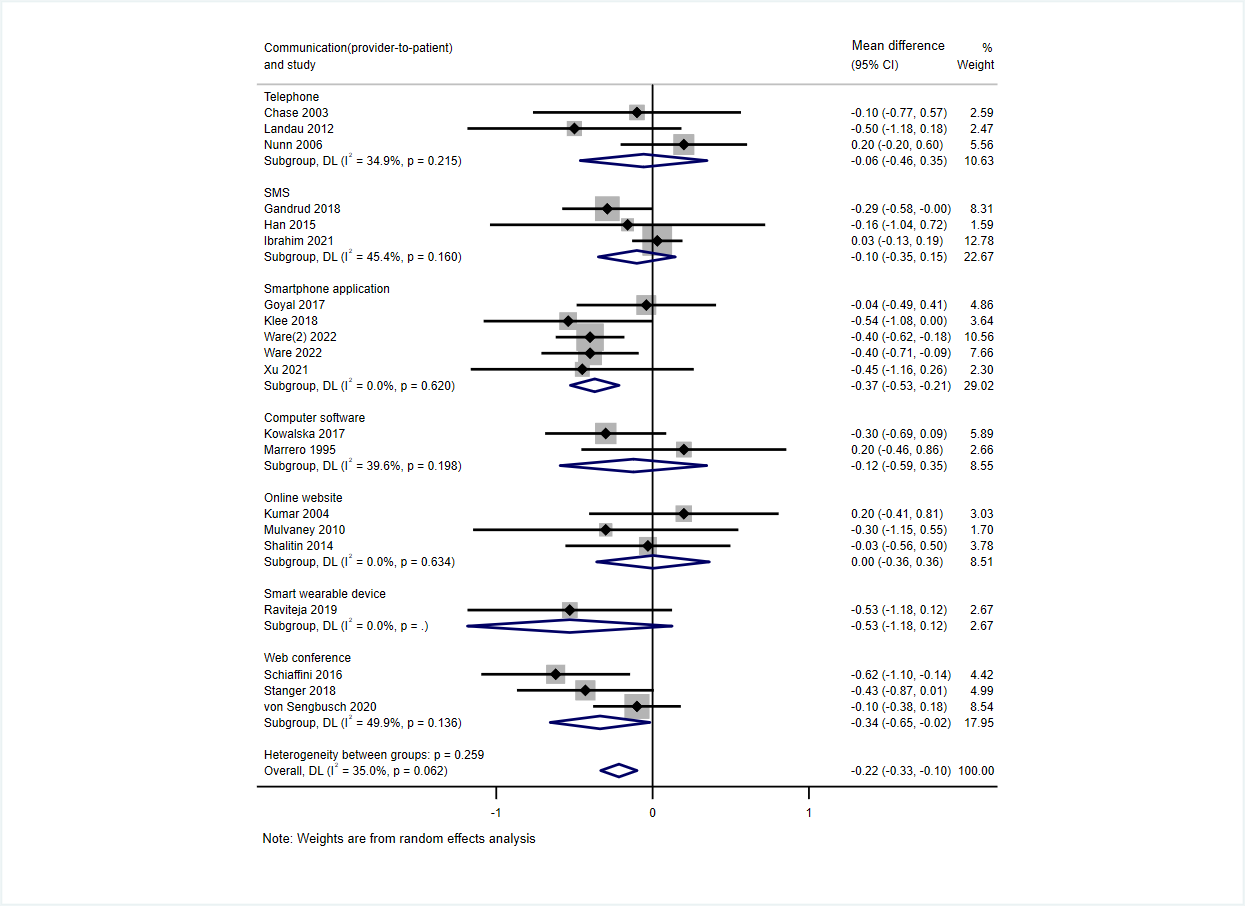
**

**G. Communication forms(patient-to-provider)**

**
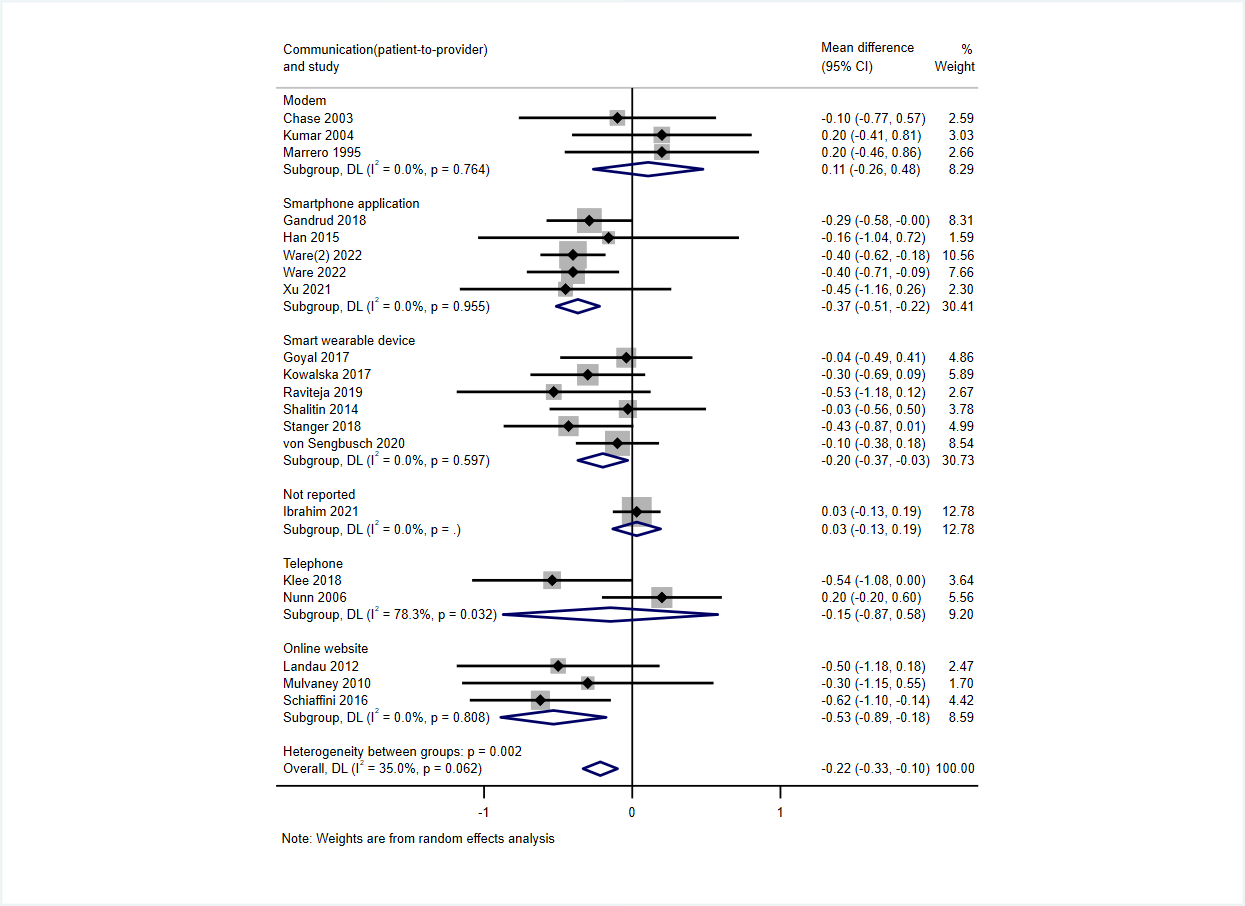
**

**H. Interactive follow-up**

**
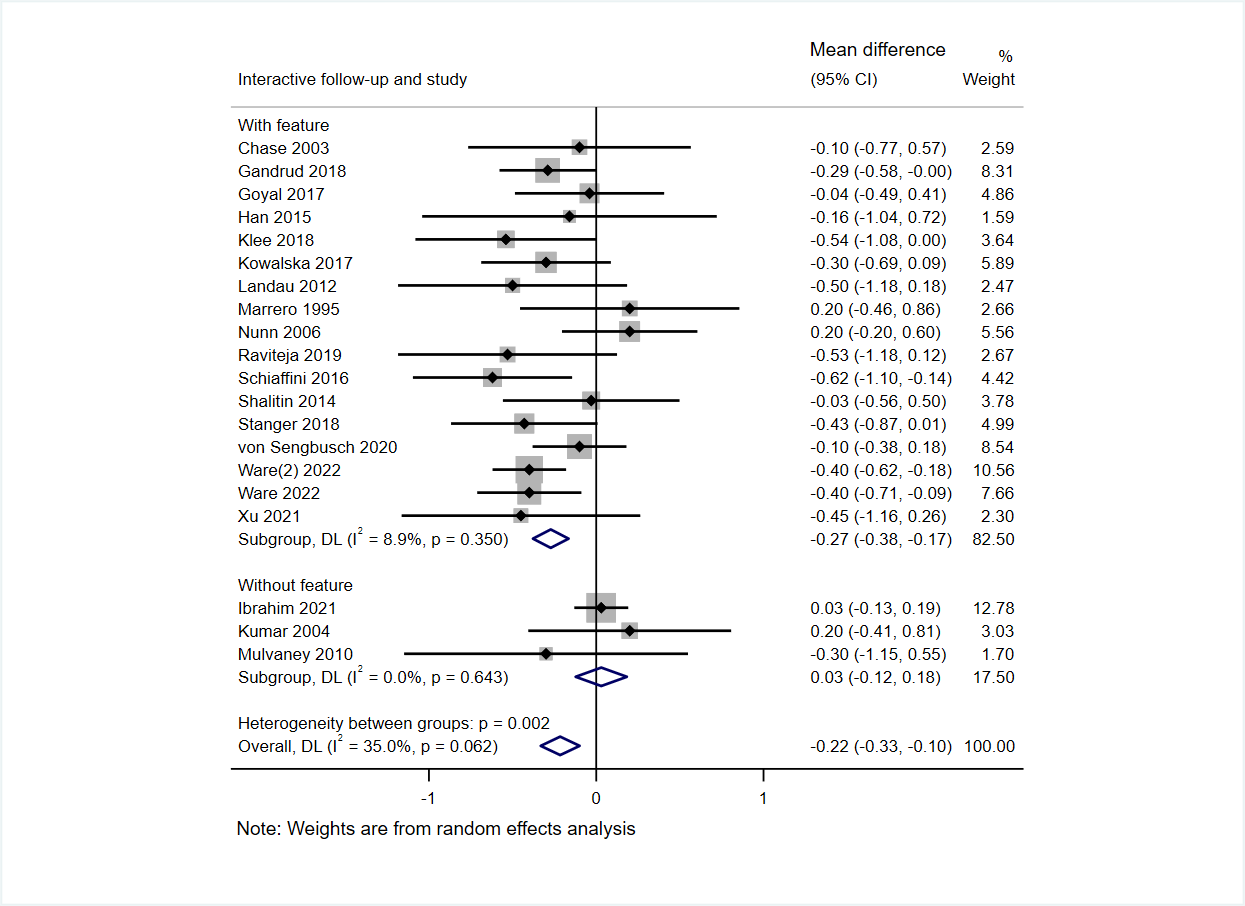
**

**I. Medication adjustment**

**
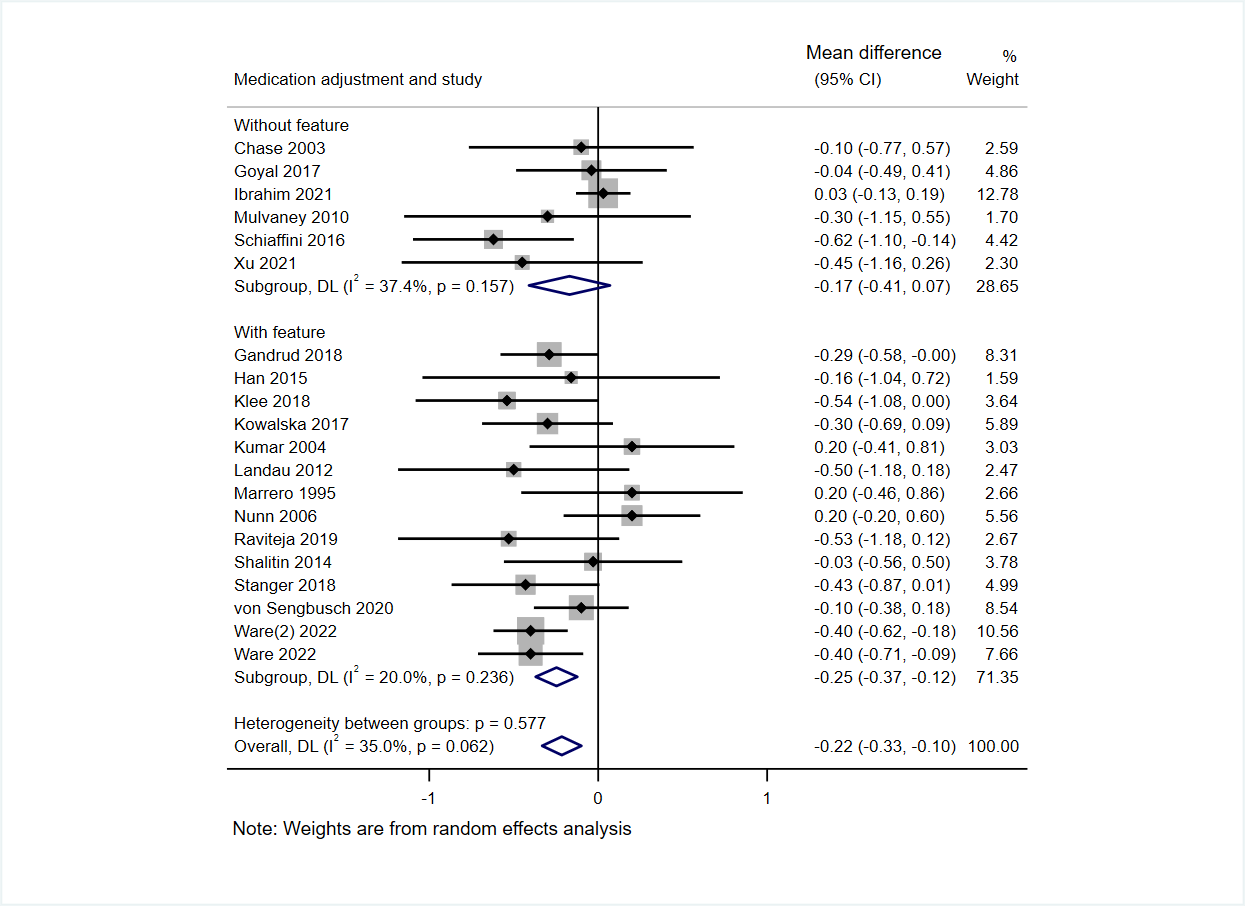
**

**J. Physical exercise**

**
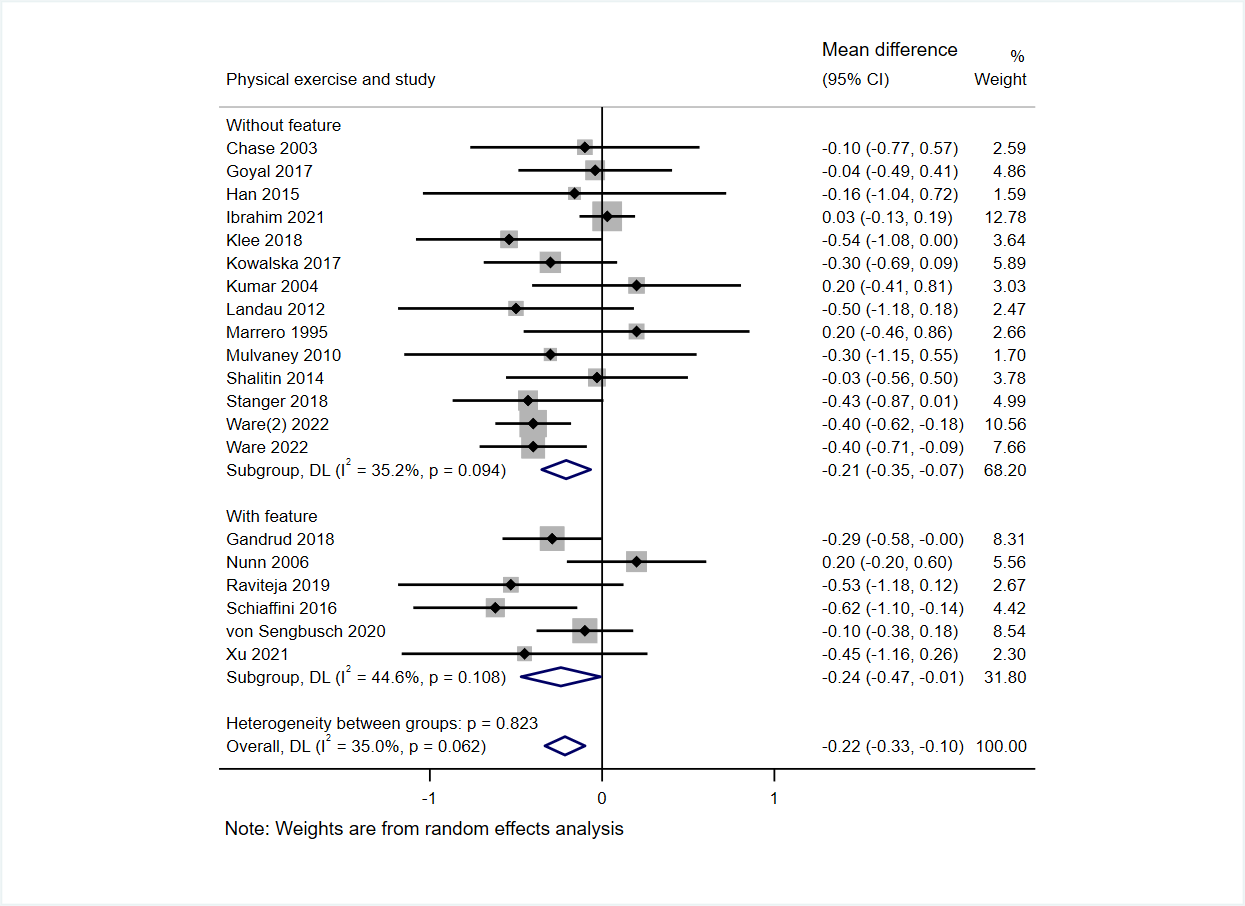
**

**K. Technology forms**

**
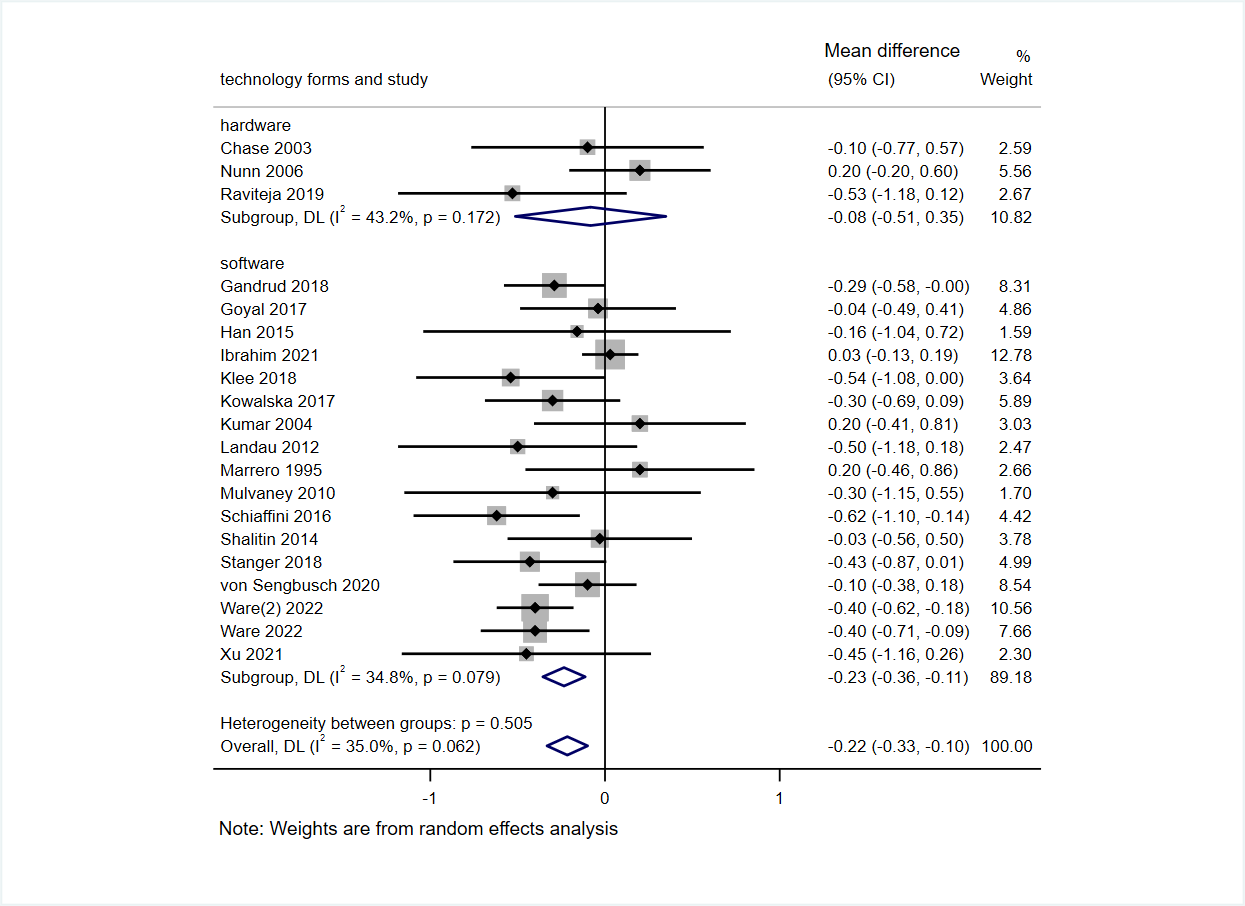
**

**L. Technology usage modes**

**
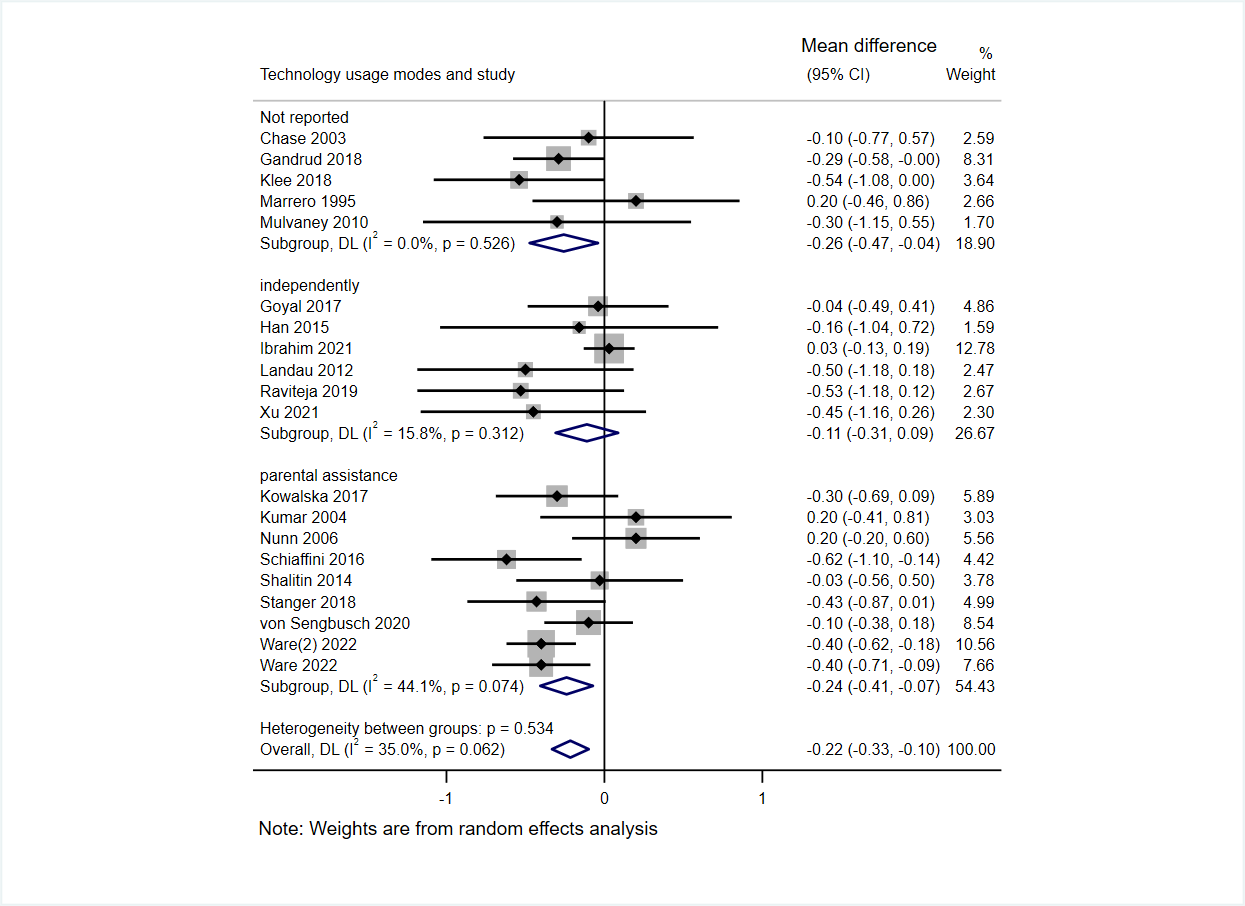
**

**M. Telemedicine intervention forms**

**
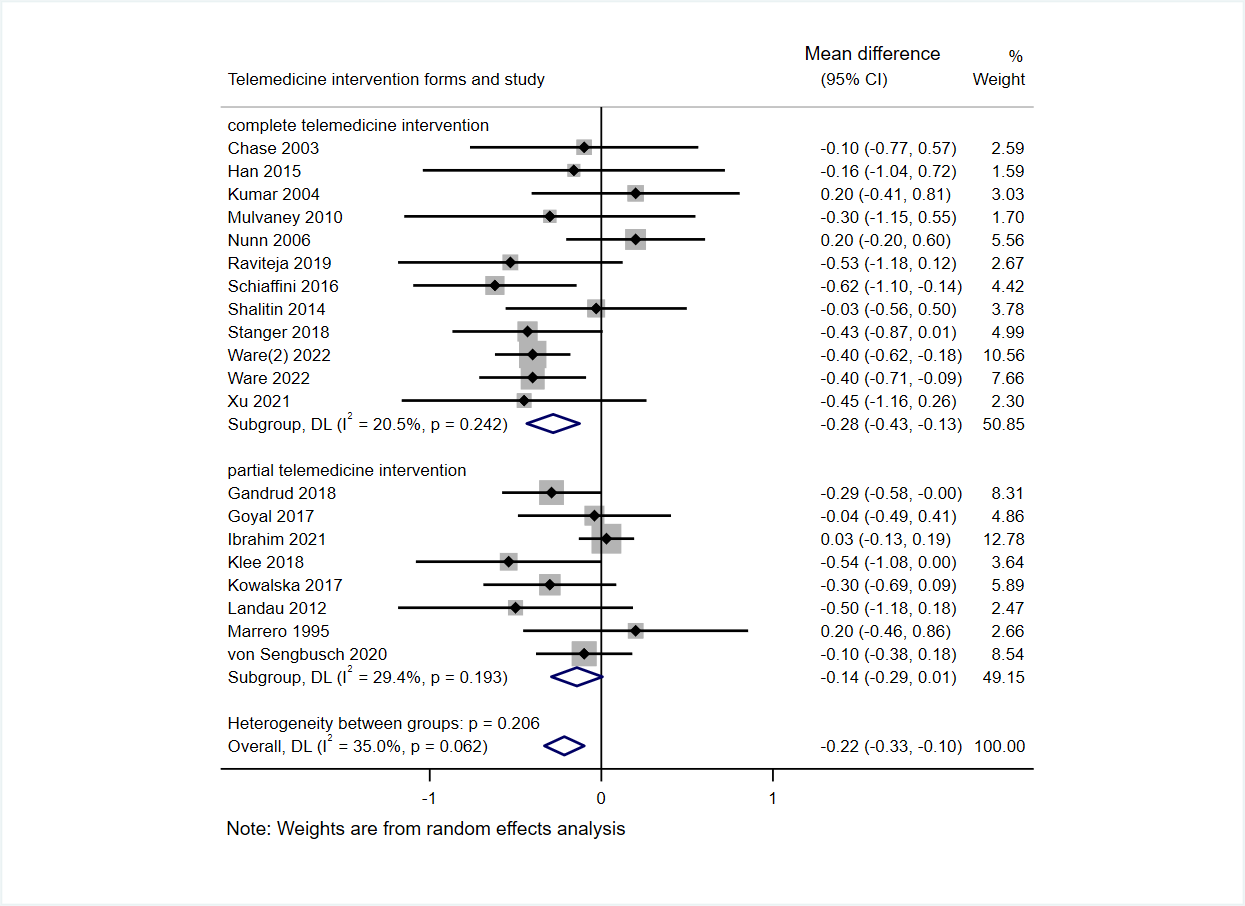
**

**Supplementary Figure S2:** Forest plots of the secondary outcomes

**A. DQOLY(Impact of diabetes)**

**
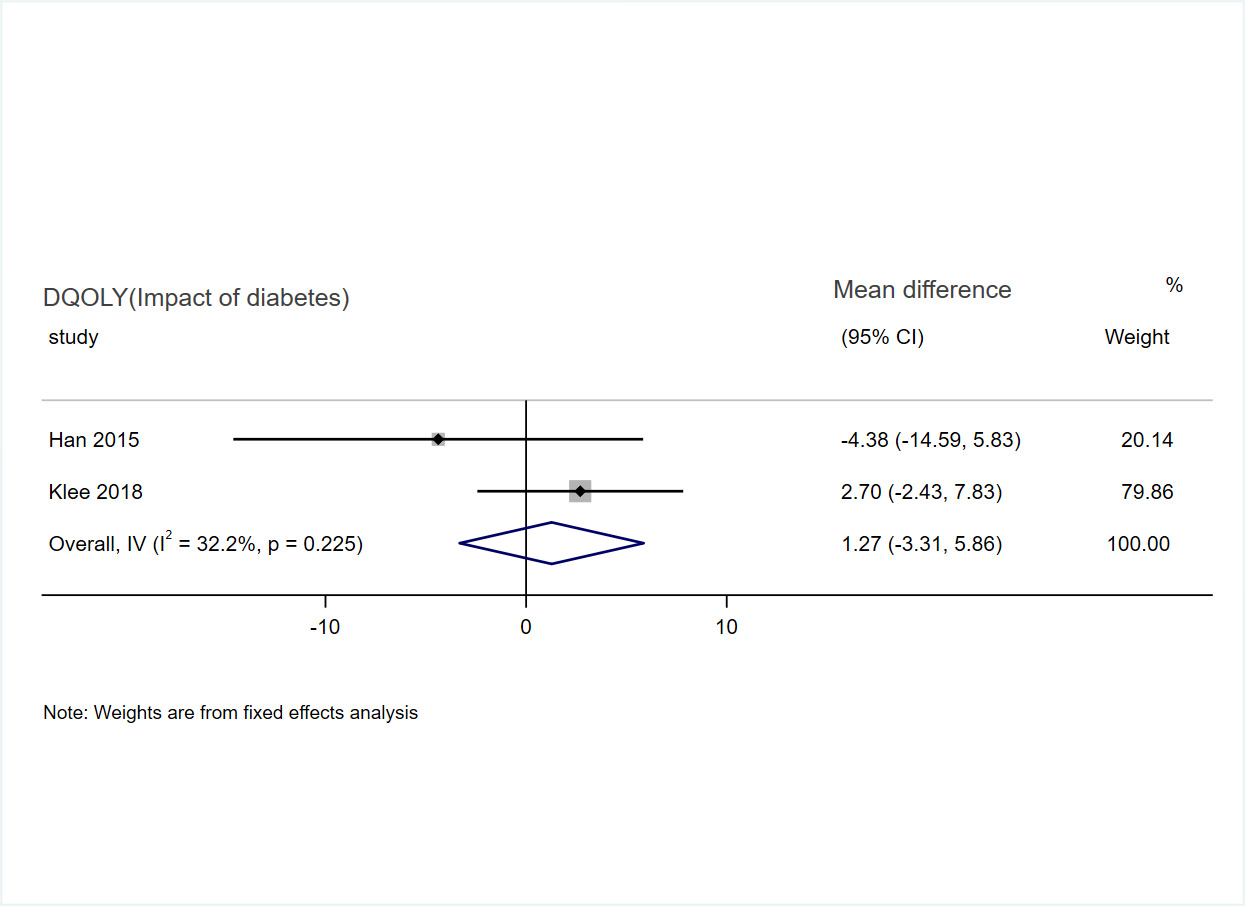
**

**B. DQOLY(Worries about diabetes)**

**
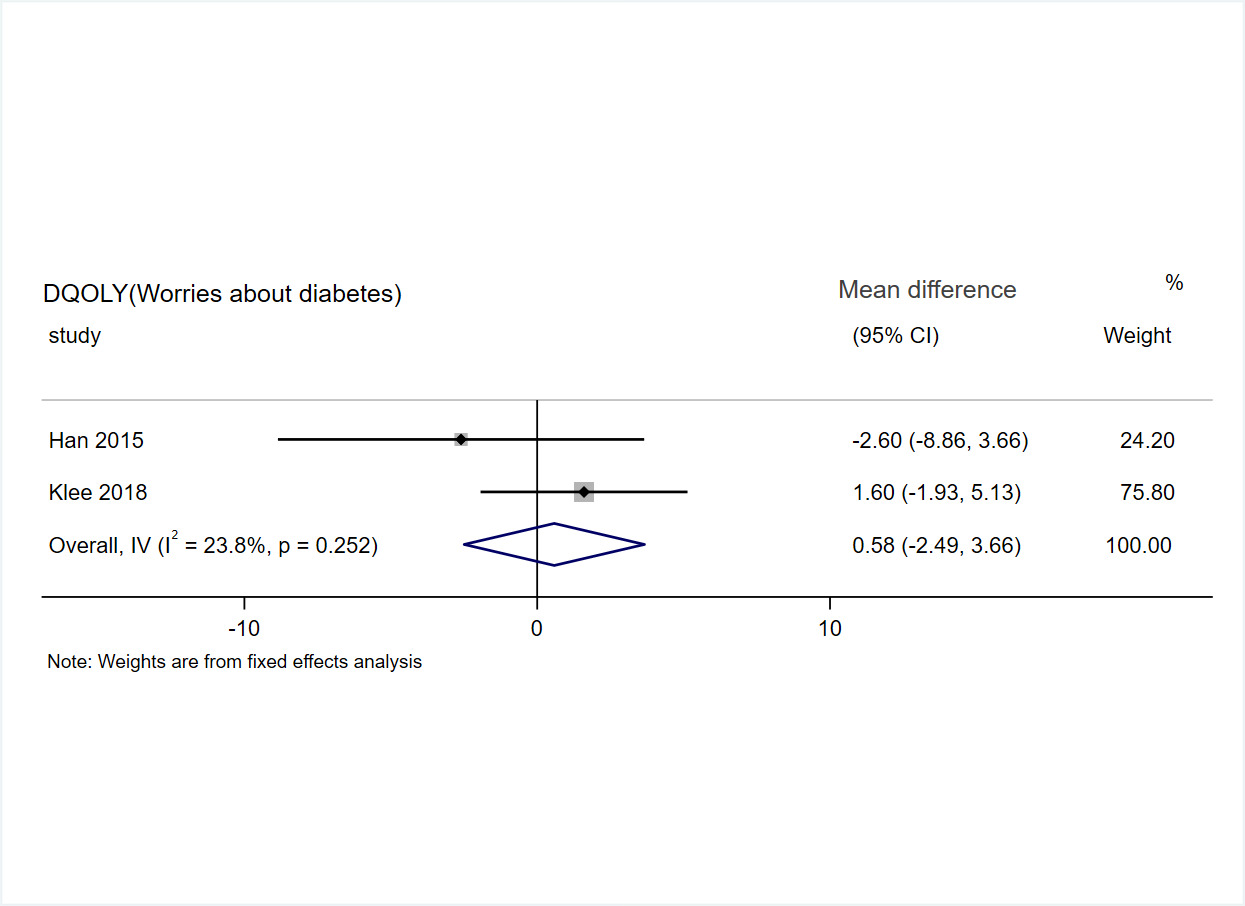
**

**C. DQOLY(Satisfaction with diabetes)**

**
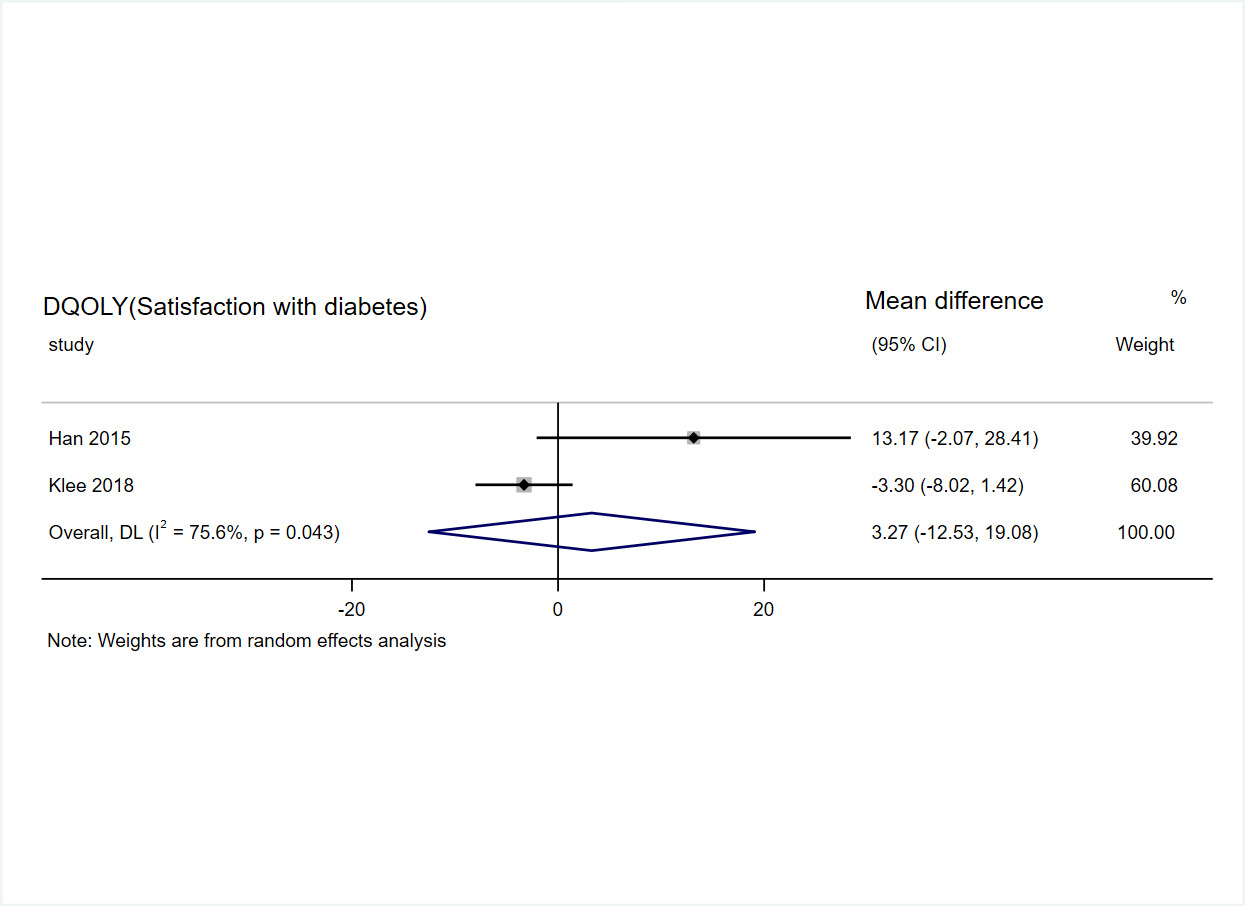
**

1. **N-QOL**

**
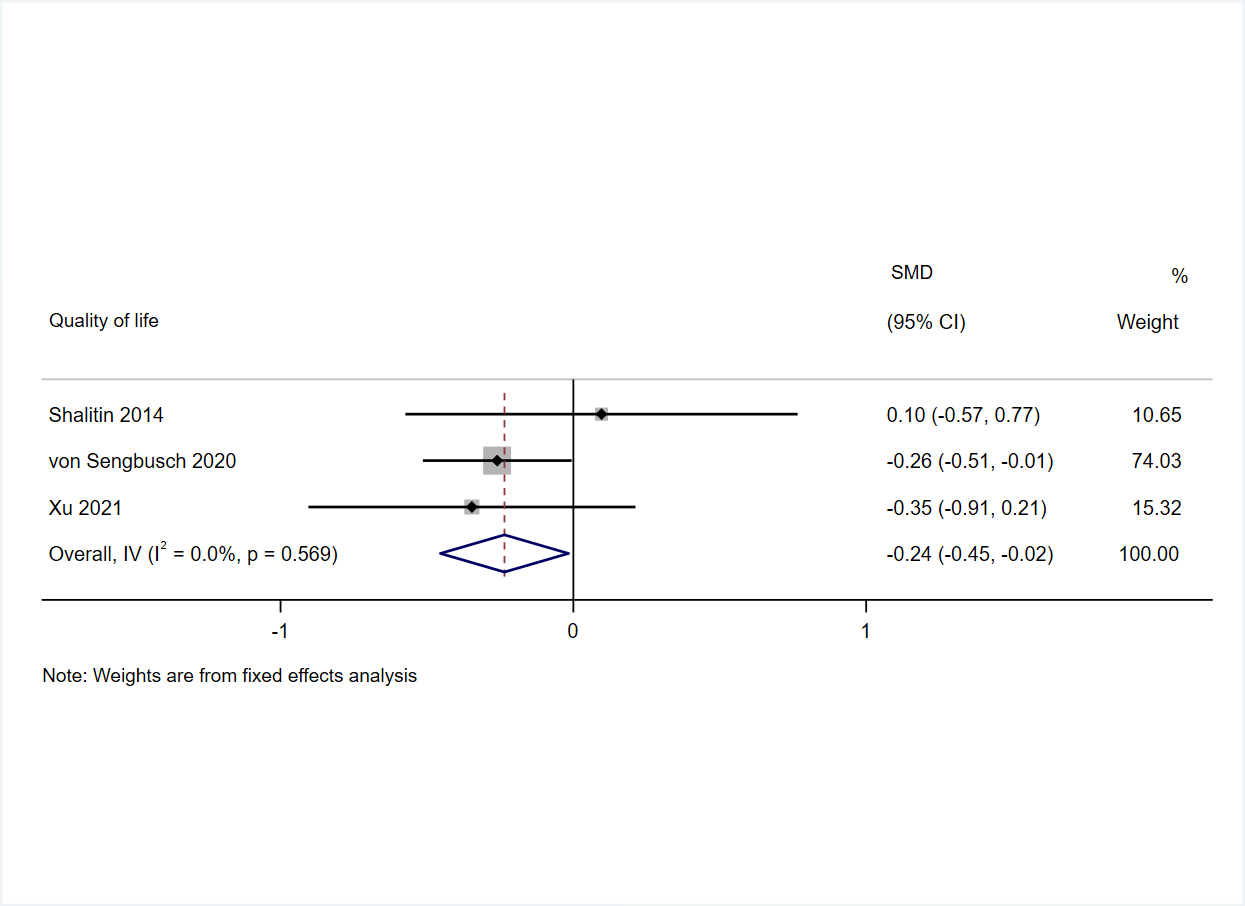
**

**E. SMBG**

**
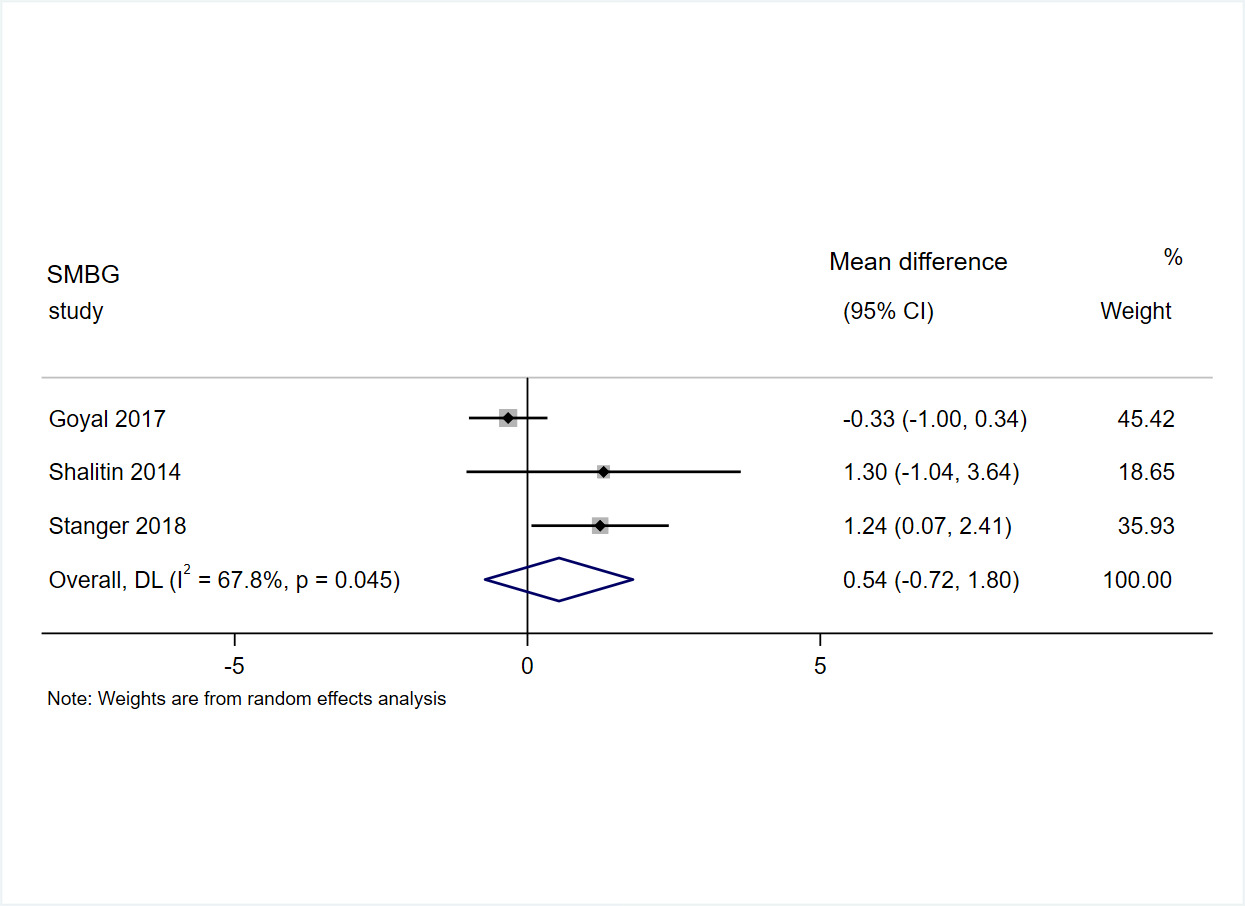
**

**F. Incidence of hypoglycemia**

**
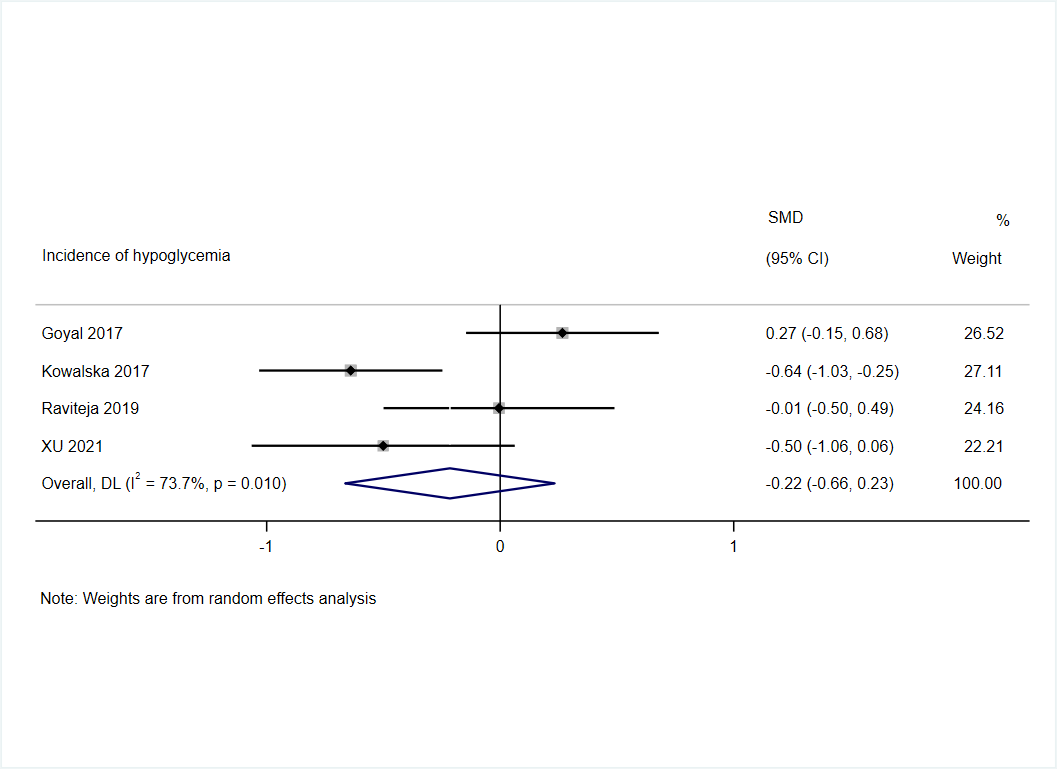
**

**Supplementary Table S1: Characteristics of the included studies**

| First author,  year,  country | RCT design | Sample size(I-C) | | Attrition | | Participants characteristics | | | | Comparator | End-point for primary outcome  (HbA1c) | Secondary outcomes | | | |
| --- | --- | --- | --- | --- | --- | --- | --- | --- | --- | --- | --- | --- | --- | --- | --- |
|  |  | After randomization | For data analysis | Overall attrition rate(%) | Attrition number and reason | Male sex(%) | Initial HbA1c(%) | Mean age(year) | Mean diabtetes duration(year) |  |  | quality of life: I(BI-AI)/C(BI-AI) | SMBG: I(BI-AI)/C(BI-AI) | incidence of hypoglycemia: I(BI-AI)/C(BI-AI) | cost: I(AI)/C(AI) |
| Chase et al[29],  2003,  America | parallel | 70(35-35) | 63(30-33) | 10.0 | n=7:  (i)Not transmitting glucose data(n=5)  (ii)Nability to attend the 3-month clinic visit(n=2) | 30(48%, 63) | ≥7.0 | 17.3 | 7.9 | Usual care (regular clinic visit every 3  months) | 6 months | NR | NR | NR | $246/$163 |
| Gandrud et al[30],  2018,  America | parallel | 117(60-57) | 117(60-57) | 9.4 | n=11:  (i)Discontinued participation early in  the enrollment period before any data were uploaded(n=4)  (ii)Discontinued participation later in the study(n=7) | 39(49%, 80) | ≥8.0 | 12.7 | 6.3 | Usual care | 6 months | DI | NR | NR | NR |
| Goyal et al[31],  2017,  Canada | parallel | 92(46-46) | 91(46-45) | 4.3 | n=4:  (i)Withdrew:  Dissatisfaction with assignment(n=1)  (ii)Discontinued:  Move away from clinic(n=2); Due to parental concern(n=1) | 41(45%, 92) | >8.0 | 14.0 | 6.9 | Usual care | 12 months | DQOLY: Worries about diabetes：I:6.83 (5.5)  -6.84 (5.8)/  C:6.51 (5.8)  -4.81 (5.0) | I:3.98 (1.6)  -3.49 (1.8)/  C:3.55 (1.6)  -3.39 (1.5) | I:10 (8.2)  -11.52 (10.7)/  C：8.49 (9.6)  -7.54 (7.7) | NR |
| Han et al[32],  2015, America | parallel | 20(10-10) | 20(10-10) | 10.0 | n=2：  (i)Lost to follow-up(n=2) | 9(45%, 20) | ≥7.5 | 13.7 | 6.6 | Usual care | 3-4 months | DQOLY:  Impact of diabetes:  I:49.10  (12.032)-  49.00(14.337)/C:44.50 (8.303)-  48.78 (10.654)  Worries about diabetes：  I:21.00 (7.211)-  19.60 (8.514)/  C:17.80  (5.827)-  19.00 (6.566)  Satisfaction with diabetes：  I:60.20 (20.335)-  66.89(10.888)/C:71.70 (9.730)-  65.22 (19.798) | NR | NR | NR |
| Ibrahim et al[33],  2021, European | parallel | 92(45-47) | 92(45-47) | 21.7 | n=20:  (i)Dropped out:  Stopped follow-up(n=5); Reason not availiable(n=9); Refuesd blood test(n=1); No need for reminders(n=3); Not suitable for teens(n= 1) | 35(38%, 92) | >9.0 | 14.8 | 8.0 | Usual care | 6 months | NR | NR | NR | NR |
| Klee et al[34],  2018, Switzerland | crossover | 55(28-27) | 33(20-13) | 40.0 | n=22:  (i)Dropped out:  A technical problem preventing the  loading of Webdia for one patient(n=9)  (ii)Non-compliant to the protocol due to insufficient use of Webdia(n=11) | 31(56%, 55) | ≥7.0 | 13.6 | 6.5 | Usual care | 3 months | DQOLY:  Impact of diabetes:  I:1.9(8.6)/  C:–0.8(6.4)  Worries about diabetes：  I:1.4(5.3)/  C:–0.2(4.9)  Satisfaction with diabetes：  I: –2.5(8.0)/  C:0.8(5.8) | NR | DI | NR |
| Kowalska et al[35],  2017, Poland | parallel | 106(53-53) | 105(52-53) | 30.2 | n=32:  (i)Dropped out:  Lost to follow-up(n=1); Started to use the ELKa toolset(n=1); Used the toolset for < 50% of meals(n=22); Did not receive or discontinued intervention  (n=8) | 42(40%，106) | ≥6.4 | 10.9 | 4.9 | Usual care | 26 weeks | NR | DI | I:0.85 (0.5)  -0.74 (0.5)/  C:0.67 (0.4)  -0.98 (0.9) | NR |
| Kumar et al[36],  2004, America | parallel | 40(19-21) | 40(19-21) | 7.5 | n=3:  Unexplained | 22(55%, 40) | ≥6.7 | 13.6 | 6.4 | Usual care | 3 months | NR | DI | NR | NR |
| Landau et al[37],  2012,  Israel | parallel | 70(36-34) | 58(24-34) | 17.1 | n=12:  (i)Did not send blood glucose levels or sent their data three times or less during the study period and were classified as non-compliant | 32(46%, 70) | ≥7.0 | 15.2 | 5.6 | Usual care | 6 months | NR | NR | NR | NR |
| Marrero et al[38],  1995, America | parallel | 106(52-54) | 106(52-54) | - | - | 63(59%, 106) | ≥7.5 | 13.3 | 6.2 | Usual care | 12 months | NR | NR | NR | NR |
| Mulvaney et al[39],  2010, America | parallel | 72(48-24) | 52(34-18) | 27.8 | n=20:  Unexplained | 40(56%, 72) | ≥7.0 | 15.1 | - | Usual care | 11 weeks | NR | NR | NR | NR |
| Nunn et al[40],  2006, Australia | parallel | 139(-) | 123(60-63) | 11.5 | n=16:  (i)Moved out of the area or refused to provide data for the study | 69(56%, 123) | ≥7.0 | 11.9 | 3.6 | Usual care | 7 months | NR | NR | NR | NR |
| Raviteja et al[41],  2019,  India | parallel | 68(34-34) | 63(30-33) | 7.4 | n=5:  (i) Lost to follow-up | 34(54%, 63) | ≥6.5 | 6.1 | 1.0 | Usual care | 3 months | NR | NR | I:0.38(5.07)/  C:0.41(6.66) | NR |
| Schiaffini et al[42],  2016,  Italy | parallel | 29(15-14) | 29(15-14) | 6.9 | n=2:  (i)Left the study during the last year of observation because of moving to  another diabetes clinic | 11(38%, 29) | ≥7.0 | 13.0 | 4.4 | Usual care | 5 years | NR | NR | DI | NR |
| Shalitin et al[43],  2014, Israel | crossover | 70(36-34) | 35(20-15) | 50.0 | n=35:  (i)Non-compliant to the protocol | 33(47%, 70) | ≥8.0 | 14.1 | 6.4 | Usual care | 4 months | DQOL：  I:0.04(0.47)/ C:0(0.33) | I: 6.9(2.9)- 8.2(4.1)/  C: 7.2(3.6)  -7.2(3.1) | NR | NR |
| Stanger et al[44],  2018, America | parallel | 61(30-31) | 61(30-31) | - | - | 35(57%, 61) | ≥8.0 | 15.0 | 6.2 | Usual care | 6 months | NR | I: 4.8 (2.8)  -5.51(0.32)/  C:4.5(2.1)  -3.97(0.32) | NR | NR |
| von et al[45],  2020, Germany | parallel | 240(120-120) | 223(112-111) | 7.1 | n=17:  (i)Participants dropped out of the study in the first 6 months | 136(57%, 240) | ≥7.0 | 10.8 | 4.4 | Usual care (regular clinic visit every 3 months) | 6 months | HRQOL  I:61.2 (22.0)  -55.0 (22.8)/  C:59.5 (23.1)  -59.3 (24.3) | NR | NR | NR |
| Ware(2) et al[46],  2022,  UK | parallel | 148(74-74) | 147(73-74) | 1.4 | n=1:  (i)Difficulties accessing trial  consumables (e.g., infusion sets, batteries, and insulin reservoirs) at the local clinic | 43(58%, 74) | ≥7.0 | 5.6 | 2.6 | Usual care | 16 weeks | NR | NR | DI | NR |
| Ware et al[47],  2022  UK | parallel | 133(65-68) | 119(57-62) | 10.5 | n=14:  (i)Withdrew after randomisation(n=10)  (ii)Withdrew before initiating treatment  with the closed-loop system(n=4), and two withdrew because of device issues | 57(43%, 133) | ≥7.0 | 12.9 | 6.5 | Usual care | 6 months | NR | NR | DI | NR |
| Xu et al[48],  2021, China | parallel | 50(25-25) | 50(25-25) | 20.0 | n=10:  (i)Lost to follow-up(n=8)  (ii)Withdrew consent(n=2) | 18(45%, 40) | ≥7.0 | 13.0 | 2.2 | Usual care | 6 months | DQOL：  I:100.95  (17.96)-  93.95(14.57)/  C：106.35  (22.16)-  106.20(22.93) | NR | I: 7.0(5.63)-  4.1 (3.46)/  C:9.25(2.9)-  8.7 (5.12) | NR |

**Supplementary Table S3:** Summary of meta-regression results

| **Variable** | **Coefficients** | **SE** | ***Z*** | ***p*** |
| --- | --- | --- | --- | --- |
| **Age** | -.1207157 | .0925313 | -1.30 | 0.208 |
| **Publication date** | -.3663339 | .1610045 | -2.28 | 0.035 |
| **Intervention duration** | -.1192745 | .1241714 | -0.96 | 0.349 |
| **Health care provider** | .0173738 | .1243379 | 0.14 | 0.890 |
| **Feedback frequency** | .0710377 | .0924541 | 0.77 | 0.452 |
| **Communication forms**  **(Provider-to-Patient)** | -.0401061 | .0318191 | -1.26 | 0.224 |
| **Communication forms**  **(Patient-to-Provider)** | -.074966 | .0366006 | -2.05 | 0.055 |
| **Interactive follow-up** | .3017143 | .1121112 | 2.69 | 0.015 |
| **Medication adjustment** | .0928504 | .1293921 | 0.72 | 0.482 |
| **Physical exercise** | .0249135 | .1329467 | 0.19 | 0.853 |

**Supplementary Figure S2:** Sensitivity analysis

**
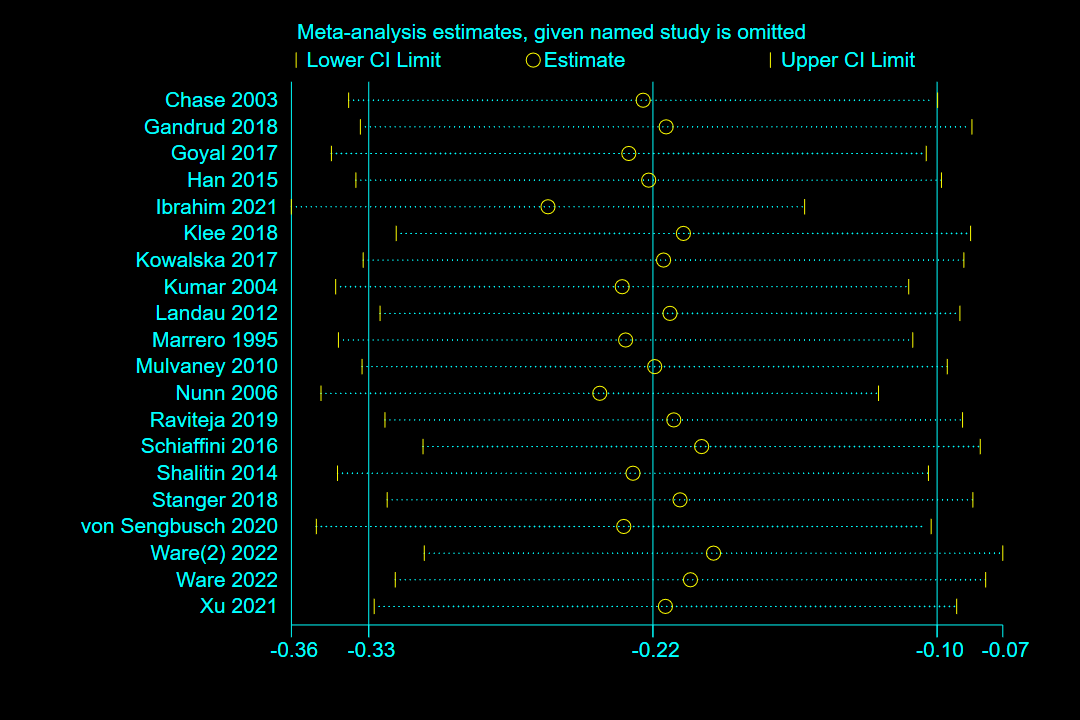
**
